# Zika, chikungunya and co-occurrence in Brazil: space-time clusters and associated environmental–socioeconomic factors

**DOI:** 10.1038/s41598-023-42930-4

**Published:** 2023-10-21

**Authors:** Raquel Gardini Sanches Palasio, Patricia Marques Moralejo Bermudi, Fernando Luiz de Lima Macedo, Lidia Maria Reis Santana, Francisco Chiaravalloti-Neto

**Affiliations:** 1 Laboratory of Spatial Analysis in Health (LAES), Department of Epidemiology, School of Public Health, University of São Paulo (FSP/USP), São Paulo, SP Brazil; 2 Epidemiological Surveillance Center (CVE) Prof. Alexandre Vranjac, Coordination of Disease Control, Health Department of the State of São Paulo, São Paulo, SP Brazil; 3 Federal University of Sao Paulo (Unifesp), São Paulo, SP Brazil

**Keywords:** Infectious diseases, Viral infection, Entomology, Climate-change ecology, Climate change, Environmental economics, Epidemiology

## Abstract

Chikungunya and Zika have been neglected as emerging diseases. This study aimed to analyze the space-time patterns of their occurrence and co-occurrence and their associated environmental and socioeconomic factors. Univariate (individually) and multivariate (co-occurrence) scans were analyzed for 608,388 and 162,992 cases of chikungunya and Zika, respectively. These occurred more frequently in the summer and autumn. The clusters with the highest risk were initially located in the northeast, dispersed to the central-west and coastal areas of São Paulo and Rio de Janeiro (2018–2021), and then increased in the northeast (2019–2021). Chikungunya and Zika demonstrated decreasing trends of 13% and 40%, respectively, whereas clusters showed an increasing trend of 85% and 57%, respectively. Clusters with a high co-occurrence risk have been identified in some regions of Brazil. High temperatures are associated with areas at a greater risk of these diseases. Chikungunya was associated with low precipitation levels, more urbanized environments, and places with greater social inequalities, whereas Zika was associated with high precipitation levels and low sewage network coverage. In conclusion, to optimize the surveillance and control of chikungunya and Zika, this study’s results revealed high-risk areas with increasing trends and priority months and the role of socioeconomic and environmental factors.

## Introduction

Chikungunya and Zika are arboviral diseases caused by viruses from the families Togaviridae and Flaviviridae, respectively, transmitted through the bites of mosquitoes of the genus *Aedes* Meigen, 1818. The number of cases related to these diseases has been increasing worldwide, expanding geographically and reaching new territories^1,2^. Chikungunya and Zika have been reported in over 116 and 92 countries (https://wwwnc.cdc.gov/), respectively, mainly in Africa, Southeast Asia, and Latin America^1,2^. The chikungunya virus (CHIKV) has spread widely since 2004, with an estimated eight million people infected worldwide. However, estimates might be underreported, as there may be approximately 100 million infections^2^. The number of Zika virus (ZIKV) infection cases has increased since 2007, with the first case confirmed in the Americas in 2014, and since 2015, the virus has been actively transmitted worldwide^1,3^.

These two infections are considered neglected tropical diseases (NTDs) and vector borne by Pan American Health Organization (PAHO)^4^. Chikungunya is recognized by the World Health Organization (WHO), but Zika has not yet been formally recognized. However, currently, chikungunya and Zika are indicated in the WHO report on NTDs in the group “Dengue and other arbovirus-related diseases”^1^. These diseases and dengue share the same vectors in the Americas, with the main one being the species *Ae. aegypti* (Linnaeus, 1762)^5^. The three diseases preferentially occur in regions with precarious socioeconomic and poor sanitation conditions, despite not being the prerogative of these areas^6^. Zika, chikungunya, and dengue are associated with the characteristics of their vectors, such as an association with deficient water supply, including still water storage, sanitary sewage, and rainwater drainage^1,7^. The co-circulation of these diseases is a matter of concern because their signs and symptoms are similar, making clinical and laboratory diagnosis difficult and posing a challenge and public health problem^8,9^.

These diseases are considered emerging and reemerging^5^ because factors such as urbanization, deforestation, and climate change, including droughts and floods, can change environments, thereby favoring their emergence or resurgence^10^. For example, ZIKV was first reported in Africa in 1947, but until 2006, it was not considered a public health problem. It emerged in 2007, with its first major outbreak occurring in 2012 in the Federated States of Micronesia in French Polynesia. ZIKV was speculated to have been first introduced into Brazil, during the soccer world cup in July 2014^11^. In February 2015, in Maranhão state, Brazil, reported cases of unknown exanthematic disease^12,13^, and Zika was subsequently confirmed. In March 2015 the first autochthonous cases were reported in Camaçari, Bahia state and Natal, Rio Grande do Norte state by RT-PCR (reverse transcription polymerase chain reaction)^11,14–16^ . This disease became notorious later, with a disproportionate increase in cases of congenital microcephaly in Brazil^3,5,11,14^. Consequently, a national health emergency was declared in 2015 by the Brazilian Ministry of Health (MH)^13,17^ and a public health emergency of international concern (PHEIC) in 2016 by the WHO^3^. The same year, Congenital Zika Syndrome was reported, which was characterized by microcephaly, neurological and neurosensory central nervous system changes, cerebral calcifications, and ocular lesions^18^.

Chikungunya is also an emerging disease. CHIKV was discovered in 1952-1953 in Tanzania, spreading to Africa and Southeast Asia and accounting for a few cases. The disease emerged in 2005 in India and Sri Lanka, with an estimated 1.4-6.5 million cases, probably because of mutations that allowed viral adaptation to a new vector, *Ae. albopictus* (Skuse, 1894)^19,20^. It was first reported in Brazil in 2014 in Amapá and Bahia and currently exists in all Brazilian states^9^. This arbovirus is more likely to cause epidemics than dengue due to shorter intrinsic and extrinsic incubation times (in the mosquito) and a longer viremia period^21^.

The incubation period of CHIKV is 3–7 days, and the main symptoms are fever and arthralgia, which can lead to death. Furthermore, 70–90% of cases are symptomatic; this percentage is higher than that of other arboviruses, which is a matter of concern because it leads to higher care demands and overloading of health services^9,21^. Patients with Zika usually have a low-grade fever and less intense arthralgia than those with chikungunya, with a mean viral incubation period of 6 days^9,22^. However, most cases are asymptomatic; however, in the most severe cases, it can affect the central nervous system and induce neurological signs associated with the onset of Congenital Zika and Guillain-Barré Syndromes in adults^23–25^.

Regarding the above, identifying risk areas for these arboviruses is important for use in advance by health services^26–30^. Previous studies identified the areas with the highest risk of chikungunya and Zika in the Caribbean, Colombia, Mexico, China, the municipality of Fortaleza, and the state of São Paulo in Brazil using spatial scanning statistics, Bayesian modeling^28–34^, Getis-Ord Gi*(d)^26,35^ or using analytic hierarchy process models^36^ and also reported a relationship between spatial clusters and socioeconomic inequality^27,30^. Cavalcanti et al.^37^ analyzed cases of ZIKV-related microcephaly in Brazil and identified clusters predominantly in Northeast Brazil, mostly in Bahia, and smaller clusters in Minas Gerais and São Paulo. In addition, to our knowledge, few studies have focused on cluster analysis of the co-occurrence of Zika and chikungunya (Table 1). Besides, modelling multiple interrelated diseases simultaneously has recently been extended^38^. In the systematic review by Tesema et al.^38^ with this approach, only two articles with Zika and chikungunya are cited. Desjardins et al.^39^, Freitas et al.^40^ and Martínez-Bello et al.^34^ studied the co-occurrence of both diseases in Colombia and Bisanzio et al.^26^ in Mexico. In addition to these, Freitas et al.^41^, Schmidt et al.^42^, Queiroz and Medronho^43^, Souza-Santos et al.^44^ and Rodrigues et al.^45^studied their co-occurrence spatial in the state of Rio Janeiro, Brazil. Pavani et al.^46^, Freitas et al.^47^, Carabali et al.^30^ and Costa et al.^27^ in other states of Brazil (Table 1). It is worth mentioning, Freitas et al.^47^, Rodrigues et al.^45^ present an alternative of co-occurrence analysis in areas with more underreporting, in regions with low income, which these used the sum of the cases of the diseases and a univariate analysis instead of a multivariate analysis.

Thus, cluster analyses of the co-occurrence of Zika and chikungunya in all Brazilian municipalities have not been conducted, and this is the focus of the present study. Therefore, even considering these diseases separately, our results present, to our knowledge, the novelty of applying scan statistics to identify clusters of the entire country, instead of inside the cities^44,48^ and states^47,49–51^. In the literature, studies on the occurrence of Zika and chikungunya involving spatiotemporal analysis and climate variations are primarily reported on a global scale. The exceptions include the studies by Dong et al.^31^ which analyzed Zika, chikungunya and Dengue in Mexico, Perkins et al.^52^, which analyzed only Zika in America, and Aguiar et al.^53^, which applied the MaxEnt method with climate variables using 2015–2016 data in Brazil, which we have updated to 2021 in our study using cluster analysis. In addition, Anjos et al.^54^, Fuller et al.^55^, Pavani et al.^46^, and Raymundo and Medronho^56^ studied these diseases on the Brasilian state scale.

This study aimed to analyze the space-time patterns of the occurrence and co-occurrence of Zika and chikungunya in Brazil. It explored the relationship among socioeconomic, demographic, and environmental variables to generate hypotheses for further testing in future studies.

## Results

### Descriptive analysis

**Figure 1:**
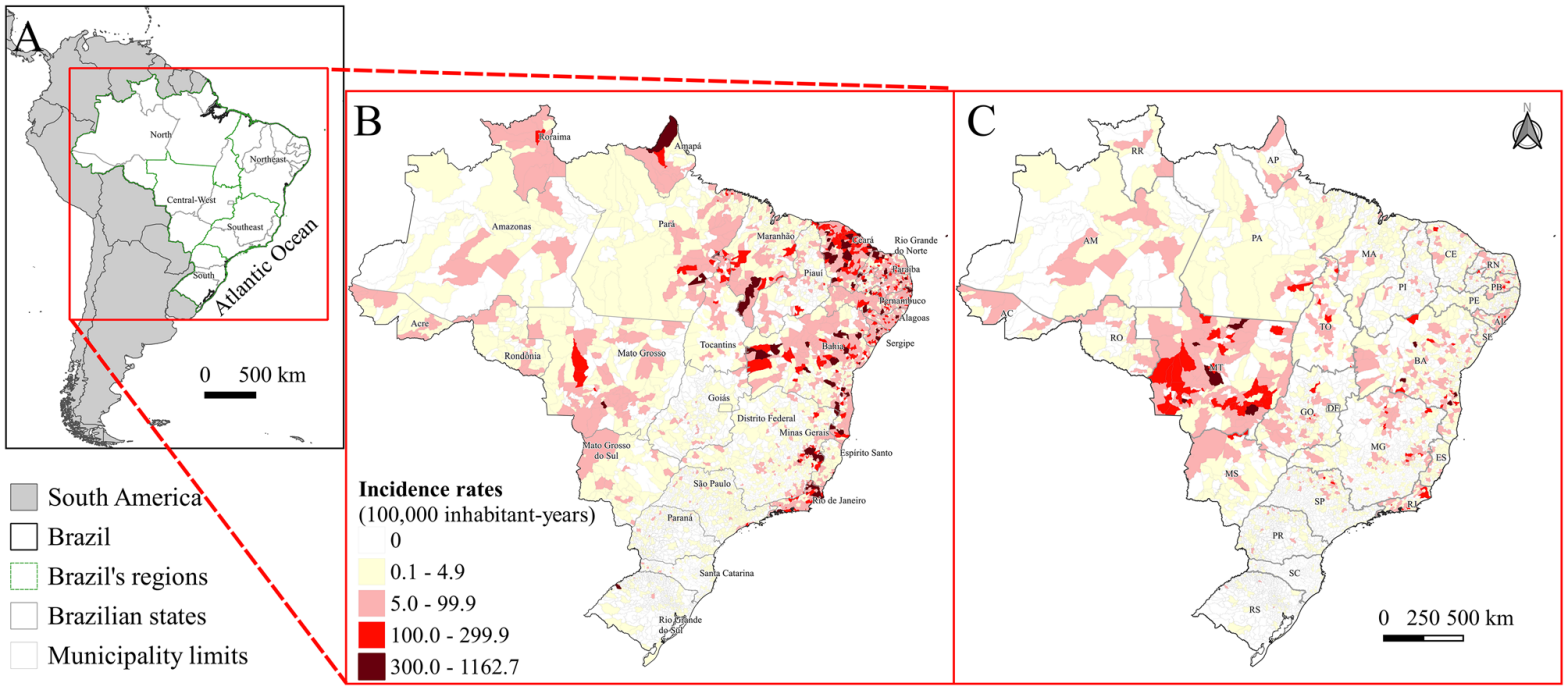
Map of South America, Brazil, and its regions (**A**), map of Brazil and its states, with the incidence rate (100,000 inhabitants-year) of confirmed chikungunya (**B**) and Zika (**C**) cases based on the municipality of residence, with symptom onset between 2015 and 2021. (**B**) Legend with the names of the states, (**C**) legend with the abbreviations of the states.

During the study period (2015–2021), 1,154,535 and 404,779 cases of chikungunya and Zika, respectively, were reported in Brazil. Of these, 528,531 and 238,462 cases of chikungunya and Zika, respectively, were excluded for the following reasons: inconclusive classification or for being under investigation (525,221; 237,371), a symptom onset date outside the study period (3308; 1079), and residing in another country (2; 12). The database had 626,004 and 166,317 cases of chikungunya and Zika, respectively, confirmed using laboratory or clinical-epidemiological criteria and distributed across 3042 and 2050 municipalities of residence, respectively (Fig. 1 and see Supplementary and Table S1a online). Of these, 850 (chikungunya) and 74 (Zika) patients distributed across 229 and 44 municipalities of residence, respectively, died during the study period (see Supplementary Fig. S1 and Table S1a and S2 online).

**Figure 2:**
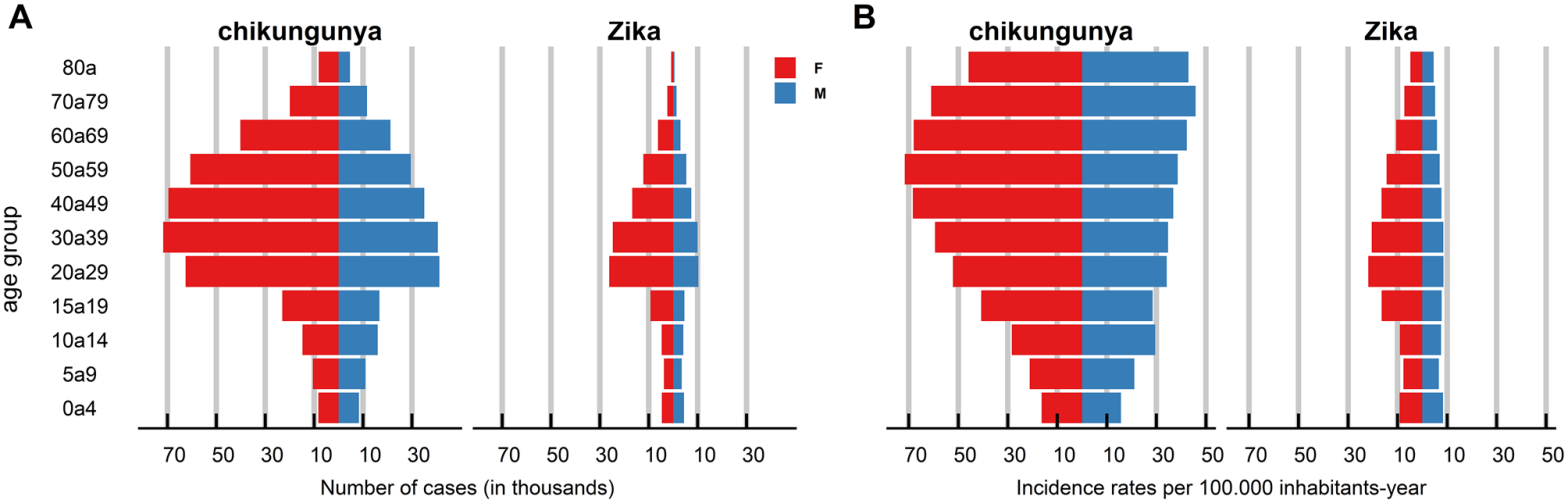
Graph of absolute numbers based on age group and sex (**A**) and incidence rates (per 100,000 inhabitants-year) (**B**) of chikungunya and Zika cases in Brazil, with symptom onset between 2015 and 2021.

The incidence rates per 100,000 inhabitants-year in Brazil were 42.90 and 11.40 for chikungunya and Zika, respectively. Incidence rates were higher among women than among men, and in the age groups>15 years for chikungunya and>10 years for Zika, with differences that persisted to approximately 79 years (Fig. 2). There was also a predominance of black and pardo race/color for chikungunya (53.73%) and Zika (37.15%). Approximately 60% of the records presented no data on the educational level (Table 2).

Univariate and multivariate scan analyses included 608,388 and 162,992 confirmed cases of chikungunya and Zika, respectively (Supplementary Table S1b and S1c Online); however, in addition to the abovementioned excluded cases, 17,110 and 3,036 imported cases and 505 and 287 cases of chikungunya and Zika, respectively, were excluded for no data on the municipality of residence and/or sex.

### Purely temporal and seasonal analysis

**Figure 3:**
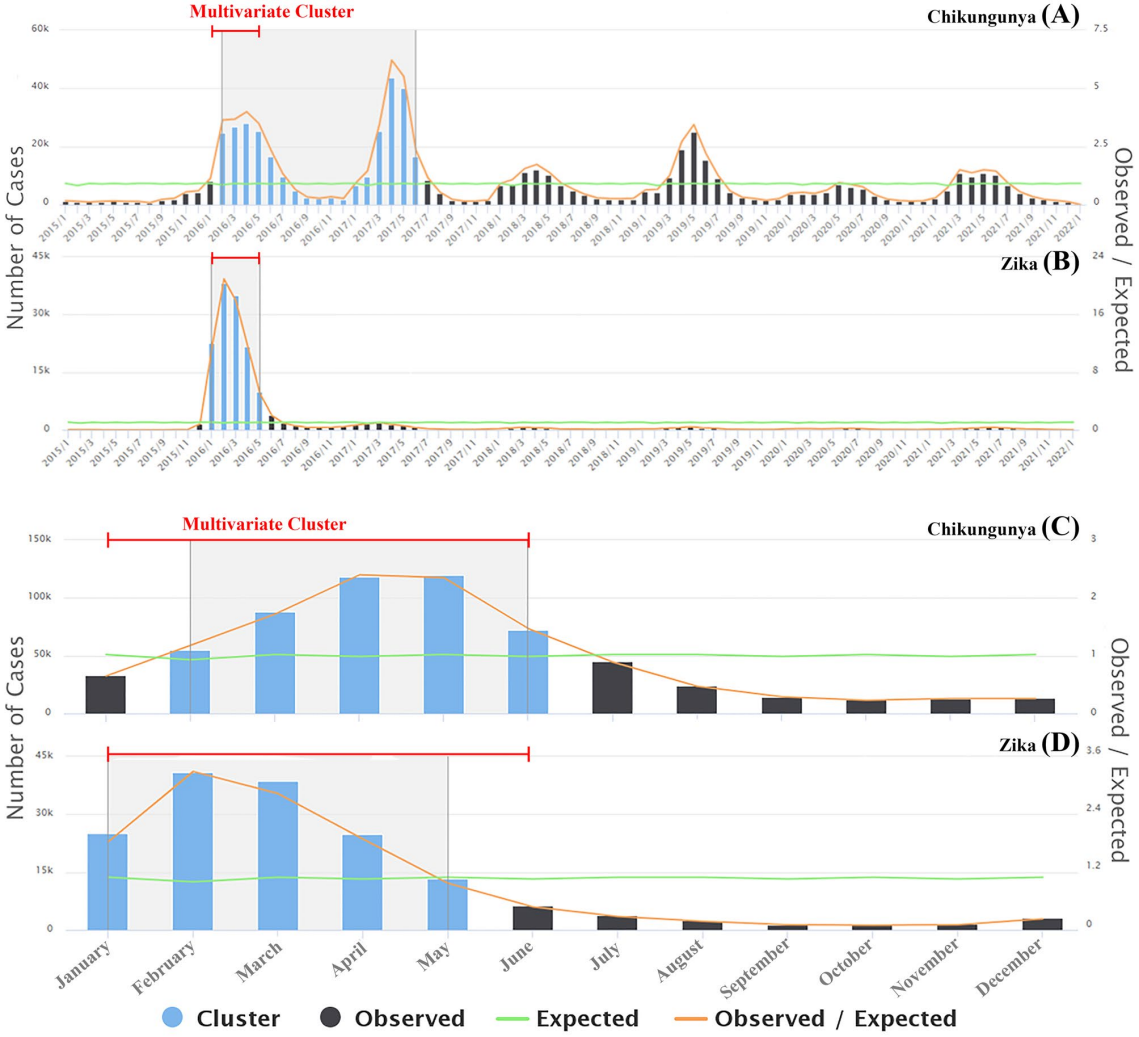
Purely temporal analysis based on month of the distribution of chikungunya (**A**) and Zika (**B**) cases, seasonal analysis of the distribution of chikungunya (**C**) and Zika (**d**) cases, and multivariate analysis (red lines) in Brazil between 2015 and 2021.

The purely temporal analysis revealed a higher risk cluster between February 2016 and June 2017 for chikungunya, with a relative risk (RR) value of 3.58, and between January and May 2016 for Zika, with an RR of 57.37. A cluster with simultaneous conditions was also identified between January and May 2016 (Fig. 3a,b). Seasonal analysis revealed a higher risk of chikungunya between February and June (summer and fall) and a higher risk of Zika between January and May, with RRs of 4.28 and 10.32, respectively. The multivariate analysis revealed that the highest risk was between January and June (Fig. 3c,d).

### Purely spatial analysis

**Figure 4:**
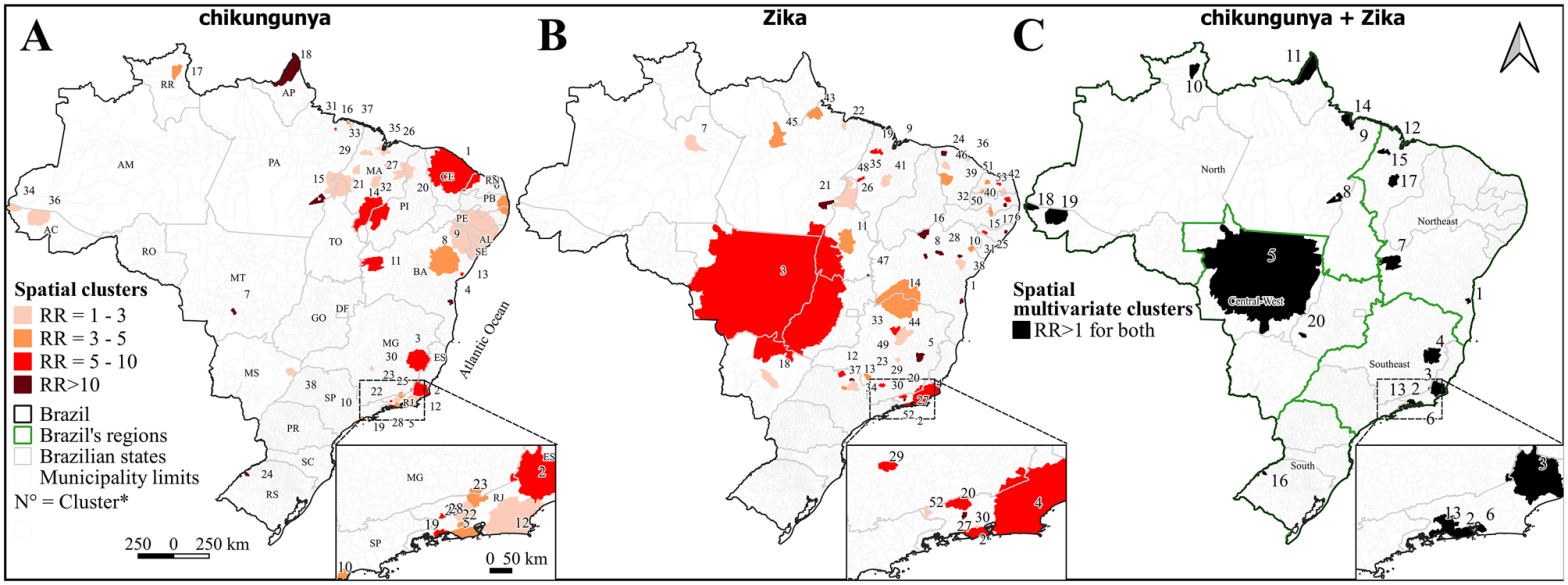
Spatial analysis of chikungunya (**A**) and Zika (**B**) cases, and multivariate (**C**) analysis in Brazil between 2015 and 2021. N = clusters’ identification number. See Supplementary Tables S3 and S4.

Univariate (Chikungunya and Zika considered individually) and multivariate (evaluating the co-occurrence of both diseases) purely spatial analyses identified 38, 53, and 20 significant clusters, respectively (Fig. 4, see Supplementary Tables S3 and S4 online). The Gini index for all the above analyses was 4% for the population of the scan windows. Chikungunya clusters were distributed across more municipalities (707) than those of Zika (520) and co-occurrence of both diseases (186).

Regarding chikungunya clusters, the Brazilian region with the most municipalities was the northeast (552), followed by the southeast (113), north (39), central-west (2), and south (1). The distribution of Zika clusters had another pattern, with more municipalities in the central-west (284), southeast (117), northeast (68), and north (21); however, no municipality was identified in the south. Co-occurring clusters were distributed across 103 municipalities in the central-west, 63 in the southeast, 11 in the north, eight in the northeast, and one in the south.

Spatial Zika clusters with the highest RR were identified in municipalities located in the state of Bahia (BA), one in Itabuna (cluster 1, RR = 118.04) and another in Uibaí (cluster 8, RR = 56.00). In contrast, the two spatial chikungunya clusters with the highest RR were identified in Itabuna and Barro Preto in BA (cluster 4, RR = 23.06) and Várzea Grande in Mato Grosso (cluster 7, RR = 15.74). In Itabuna and Barro Preto in BA, co-occurrence analysis also revealed the cluster with the highest RR for chikungunya and Zika (cluster 1, RR = 23.06 and 115.69, respectively) (Fig. 4, see Supplementary Tables S3 and S4 online).

### Space-time analysis

**Figure 5:**
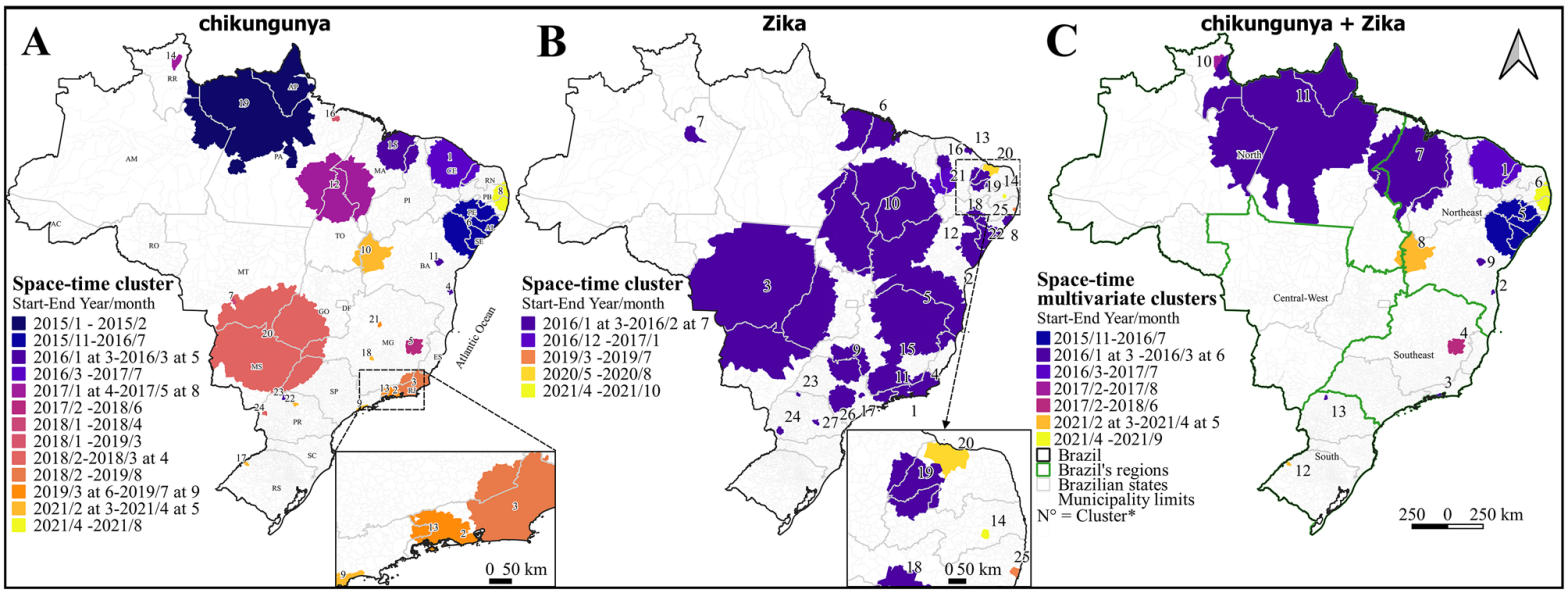
Space-time analysis of chikungunya (**A**) and Zika (**B**) cases, and multivariate (**C**) analysis in Brazil between 2015 and 2021. N = clusters’ identification number, see Supplementary Tables S5 and S6.

The space-time analysis for chikungunya, Zika, and co-occurrence revealed 24, 27, and 13 high-risk clusters, respectively. This analysis showed that most clusters began between January and April throughout the study period, except for chikungunya (cluster 6) and co-occurrence (cluster 5), which began in November 2015, chikungunya (cluster 21) in June 2019, and Zika (cluster 21) in December 2016 and (cluster 20) in May 2020 (Fig. 5, see Supplementary Tables S5 and S6 online). For chikungunya, clusters that started in 2015–2017 predominated in the north and northeast regions, whereas in other regions, the clusters were identified predominantly between 2018 and 2021. However, the most recent cluster was located in the northeast region between April and August 2021 (cluster 8, RR = 17.08) (Fig. 5). For Zika, clusters that started in 2016–2017 predominated in all regions of Brazil, except for three clusters that occurred between 2019 and 2021 in Pernambuco (cluster 25), Roraima (cluster 20), and Paraíba (cluster 14). In multivariate analysis, clusters that started between 2015 and 2017 predominated, except for three clusters that started in 2021 in the northeast: Paraíba and Pernambuco (cluster 6) and Bahia (cluster 8), and in the south: Rio Grande do Sul (cluster 12) (Fig. 5, see Supplementary Tables S5 and S6 online).

### Spatial variation analysis in temporal trends

**Figure 6:**
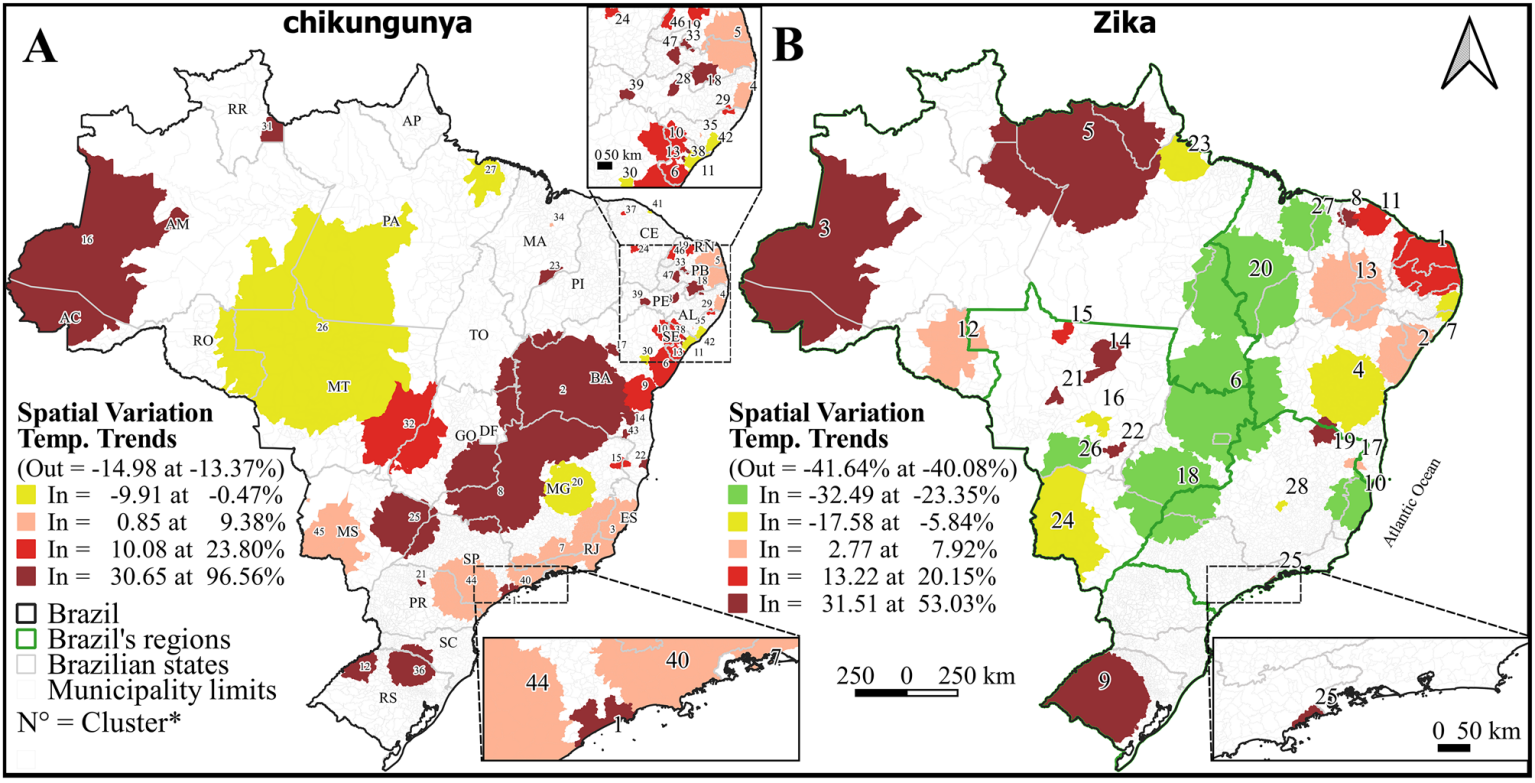
Spatial variation analysis in temporal trends of chikungunya (**A**) and Zika (**B**) cases in Brazil between 2015 and 2021. N = clusters’ identification number, see Supplementary Table S7. Out = Outside the cluster. In = Inside the cluster.

Spatial variations in the temporal trends for chikungunya and Zika decreased across the country by 13% and 40%, respectively, with 47 and 28 significant clusters identified for chikungunya and Zika, respectively. Despite the decreasing temporal trend of chikungunya throughout the country, spatial variation clusters with a growing internal trend predominated in practically all states, with annual growth of 0.85%–96.56%. Only seven of the 47 clusters of chikungunya demonstrated a decreasing trend. Spatial variation analysis of temporal trends for Zika identified 16 clusters with an increasing annual trend and 12 with a decreasing trend, with internal trends ranging from −32.96% to 53.03%. Clusters with annual increases were located in the north and northeast, in the states of Rio Grande do Sul, Mato Grosso, Minas Gerais, Bahia, and Espírito Santo, and on the coast of São Paulo (Fig. 6, see Supplementary Table S7 online).

### T-test of socioeconomic and environmental variables

The t-test for statistical comparison of socioeconomic and environmental variables between municipalities included in purely high-risk spatial clusters and those not included showed significant differences between groups. Municipalities included in high-risk clusters for both diseases had higher temperatures than those not included.

Municipalities included in high-risk clusters for chikungunya had lower precipitation levels and a lower percentage of households with a sewage system or septic tank, running water, and garbage collection by cleaning services than those not included. These municipalities also had lower Normalized Difference Vegetation Index (NDVI) values in urban areas and higher Brazilian Deprivation Index (BDI) measurements.

In contrast, Municipalities included in high-risk clusters for Zika had higher precipitation levels and a lower percentage of households with a sewage system or septic tank than those not included. These municipalities had lower BDI measurements, a higher percentage of households with garbage collection by cleaning services, and lower NDVI values in urban areas. All analyses resulted in significant *p* values, except for the percentage of households served piped water for Zika (Table 3, see Supplementary Fig. S2 online).

## Discussion

Scan statistics were used to identify high-risk areas for chikungunya, Zika, and their co-occurrence in Brazil between 2015 and 2021. The results were consistent with those of previous studies, indicating northeast Brazil as the region with the highest incidence of these diseases between 2015 and 2016^53,57,58^. These risk areas remained active in 2017 for chikungunya. The results also showed a decline in the incidence of both conditions in Brazil between 2018 and 2021.

The spatial and temporal patterns of the two arboviruses have been changing, with the dispersion of their occurrence from the northeast to the central-west region, a change already reported in the literature for Zika^58,59^. A resurgence in the occurrence was identified in the northeast between 2019 and 2021 for Zika and in 2021 for chikungunya. Dispersion and an upward trend were also observed for chikungunya along the coast of São Paulo, especially in Cubatão, Guarujá, Santos, and São Vicente in 2021^60^ and in Rio de Janeiro between 2018 and 2021. However, some hypotheses for these pattern changes include the degree of susceptibility of populations in different Brazilian regions^58,61^ and climate change associated with greenhouse gas emissions^62^. For example, the highest temperature increase in the state of São Paulo over the last few decades was observed in municipalities located on the São Paulo coast^63^.

Seasonal scan analysis revealed that the greatest risk of occurrence of these diseases in the first months of the year was during summer when higher temperatures occur in the Brazilian regions, consistent with the results of our statistical analyses. This was related to the increased *Ae. aegypti* infestation levels due to the decreased time for larval development and increased proportion of infectious mosquitoes, given the decreased intrinsic incubation periods of the viruses in the vector^58,64^. Studies conducted in China, the United States, and the states of Rio de Janeiro and São Paulo also indicated that temperature influenced the distribution patterns of *Ae. aegypti* and *Ae. albopictus*, consequently influencing the incidence of diseases they transmit^65–69^.The small but significant differences (from 0.7 to 2.6 $^\circ$C) in the average temperature between the high-risk and no-risk areas for both diseases are worth consideration. Banu et al.^70^ showed that an increase of 1 $^\circ$C could be related to a future rise in arbovirus cases.

In recent decades, consistent and widespread warming has been observed throughout Brazil, with greater extreme heat occurring during spring and summer^71^. Increased temperatures are also expected in the coming years in Brazil, mainly in the Amazon, Tocantins, and Paraná River basins, based on future climate change scenarios and considering increased greenhouse gas levels^72^. This may indicate a greater risk of the occurrence of arboviruses in the future.

Furthermore, several factors may be associated with the inverted results obtained for precipitation behavior in clusters at high risk of chikungunya and Zika. One of the hypotheses is that the risk may increase after extreme conditions, either in places with a lot of drought or rain, as reported in dengue cases in Brazil^73^. In addition, these authors related extreme precipitation levels to urbanization and socioeconomic factors. They concluded that the risk of dengue was higher under extremely wet conditions in more rural areas and after extremely dry conditions in highly urbanized areas with a higher frequency of water shortage^73^.

This is consistent with the present study because the areas at high-risk of chikungunya were more socioeconomically unfavorable and had lower precipitation levels and a lower percentage of water supply. Lower proportions of households with water supply, sewage systems, garbage collection, and higher BDI values (a larger percentage of the population with per capita income below half of the minimum wage, illiterate people, and inappropriate households) in high-risk clusters for chikungunya indicate that these are vulnerable areas from a socioeconomic point of view^74^. In addition to the lower levels of precipitation identified in these clusters, these could lead to increased breeding sites and an abundant mosquito population owing to inadequate water storage and waste disposal, thereby raising the incidence of the disease^1,7,53^. The risk of Zika in places with higher precipitation levels, as highlighted by Lowe et al.^73^ regarding dengue, also indicates that these areas are suitable for developing *Aedes* by providing an increased number of artificial and/or natural breeding sites.

Despite the significant difference, the similarity between BDI means for areas at high-risk of Zika and other diseases may indicate that socioeconomic variables had little influence on their distribution in the Brazilian territory. The differences between worse sewage collection indicators and better garbage collection indicators in areas at risk of Zika may be associated with a large proportion of municipalities in high-risk clusters located in the central-west region of Brazil, a region that, despite having good piped water and garbage collection coverage, has precarious sewage system coverage^75,76^. According to the 2017 Brazilian National Basic Sanitation Survey (PNSP), the central-west has, second to the north region, the lowest proportion of municipalities with sewage system services (38.1%)^76^.

The positive relationship with garbage collection in municipalities with high-risk clusters for Zika can be a confusing factor. Municipalities have cleaning services; however, this does not assure proper solid waste disposal, which may end in dumping grounds (open pits). In 2008, the central-west, north, and northeast regions ofBrazil had the highest proportions of municipalities with this type of disposal. In addition, the central-west and northeast regions had the highest proportion of municipalities with waste pickers in dumps or landfills at 46.6% and 43.1%, respectively^75^. These conditions increase the number of possible vector-breeding sites in urban areas.

Brazil has continental dimensions, with social inequalities, precarious socioeconomic levels, heterogeneous basic sanitation services, and climatic differences between the five regions, mainly regarding precipitation. A study that analyzed the Brazilian regions’ seasonal and climatic trends between 1961 and 1981 reported that the northeast region underwent changes during a drier climate, mainly in the summer. In the southern region and southern parts of the central-west region, the climate became more humid; however, there were no significant changes in the other regions during this period^71^. Therefore, in addition to studies considering climate change scenarios with greater greenhouse gas emissions, more studies should be conducted at different geographic scales^58^.

A lower amount of vegetation in the urban area was statistically significant for chikungunya and Zika risk areas; nonetheless, their average values were similar. High-risk areas for Zika were associated higher precipitation levels, which could be associated with the greater presence of *Ae. albopictus* in these areas. This vector is most likely found in colder and wetter suburban and rural areas36; therefore, this could be a hypothetical explanation for the differences between diseases. *Ae. albopictus* naturally infected with ZIKV has already been reported in Brazil^77^. In addition, rapid index surveys for Aedes (LIRA) have shown that *Ae. albopictus* expanded its geographic distribution in Brazil between 2015 and 2020 and was registered in 37.4% of the surveyed municipalities. The central-west region demonstrated the most significant increase in this species among all the Brazilian regions^78^. However, previous studies reported that *Ae. aegypti* has a higher transmission rate and is more easily infected by ZIKV than CHIKV^79^. The interactions between the vectors (*Ae. albopictus* and *Ae. aegypti*) and viruses are subject to constant mutations; consequently, vector competence may change over time^59^.

The absence of a perfect overlap between spatial and space-time clusters for Zika and chikungunya in the present study is consistent with the results of other studies^41,59,80^. Differences between the risk areas for both diseases could be associated with the abovementioned factors. However, simultaneous risk areas for both diseases stand out, such as Itabuna, BA, a place with a high incidence of dengue^81^.

This study had some limitations, including the use of socioeconomic data from the 2010 census and PNSP data on solid waste, which was updated in 2008, and the use of secondary data on Zika and chikungunya, with a significant percentage of cases confirmed using clinical-epidemiological criteria. The MH recommends these criteria, which are used after confirmed sustained transmission in a certain geographic area^9^.

In conclusion, this study’s results are useful for selecting areas at greatest risk, areas with increasing trends, and the months with the highest incidence, to optimize surveillance and control actions for Zika and chikungunya. This is necessary, considering the high costs associated with arboviruses (dengue, chikungunya, and Zika). In Brazil, costs for combating the vector and medical costs (direct and indirect) accounted for approximately 2% of the planned budget for the entire health area in 2016, showing a relevant impact on society^82^. The indirect costs of productivity loss during periods of medical leave for chikungunya and Zika accounted for a reduction of approximately 429 and 48 million dollars (mean BRL/USD exchange rate for the year 2016), respectively, compared with the 2016 GDP. The direct medical costs reached USD 291 million for both diseases^82^. This indicates that directing and optimizing the implementation of surveillance and control measures can provide a more rational use of available resources. It is also important to consider socioeconomic, basic sanitation, and climatic factors in decision-making. These can also help implement surveillance and control activities to avoid or minimize analyzed diseases.

## Methods

The study area covers the Brazilian territory and its 5570 municipalities as units of analysis, which were grouped into five regions (Fig. 1), with a territorial area of 8,510,820,623 km² and an estimated population of 213,317,639 people in 2021^83^.

Data on the date of symptom onset, patient age, sex, race/color, disease progression, municipality of residence, classification, epidemiological profile, and confirmation criteria for the Zika and chikungunya cases notified in Brazil between 2015 and 2021 were obtained from the databases of the Notifiable Diseases Information System (SINAN) using the website of the Department of the Unified Health System of the Brazilian MH^84^. The spatial analysis included cases with laboratory and clinical-epidemiological confirmations. Imported cases, cases with no information on sex, and patients not residing in Brazil were excluded from the scan analyses in this study.

Incidence and mortality rates (per 100,000 inhabitants-year) were mapped by the municipality of residence between 2015 and 2021 and by each condition using Quantum Geographic Information System (QGIS) software version 3.22^85^. In addition, graphs (based on age group and sex) were created for the absolute numbers and incidence rates of each disease in Brazil during the study period using R software version 4.1.0^86^. Estimates considered the mid-period population (2018) obtained from the Brazilian Institute of Geography and Statistics (IBGE) and cartographic materials (municipal and regional meshes)^87,88^.

High-risk areas for the occurrence of chikungunya and Zika were identified based on scan statistics; relative risks (RR), which correspond to the ratios between the incidence rates inside and outside the clusters, were obtained. Three tables were built: a) chikungunya and Zika cases based on the municipality of residence, age group (classified into 11 groups: 0–4, 5–9, 10–14, 15–19, 20–29, 30–39, 40–49, 50–59, 60–69, 70–79, and >= 80 years), and sex for each symptom onset date; b) 2018 population based on age group (11 groups described above) and sex; and c) centroid coordinates of Brazilian municipalities^88^. Scan statistics were performed using the SatScan software version 10.0.02^89^.

Univariate scan analyses were performed for chikungunya and Zika cases (considered individually), and multivariate analyses were performed for both diseases (together) to assess co-occurrence. These analyses compared the number of observed and expected cases inside and outside possible clusters in multiple window sizes. The windows are circles (in the space and spatial variation in temporal trend analysis) or cylinders with a circular base and a time interval as the height (in the space-time analysis). The expected cases were obtained through an indirectly standardized method considering sex and age^90^. The SaTScan considers, for the analyses carried out in this study, the discrete Poisson model, where the number of cases in each location is Poisson-distributed. This probability distribution is well-suited for analyzing event count data, such as disease occurrences. This model was used under the following conditions: circular shape clusters, no geographical overlapping, adjustment for age and sex, and using the Monte Carlo method with 999 repetitions to estimate probabilities. Purely spatial, purely temporal, seasonal, and space-time analyses were performed to find high-risk rates, and spatial variation in temporal trend analysis was performed to find clusters with high and low temporal trends^90^. The RR for each cluster is the estimated risk within it divided by the estimated risk outside it, as presented in the following formula^90^:

$$\begin{aligned} RR = \frac{c/ E[c]}{(C-c)/(E[C]-E[c])} = \frac{c/E[c]}{(C-c)/(C-E[c])} \end{aligned}$$

where, C and E[C] are respectively, the observed and expected number of total cases, and c and E[c] are, respectively, the observed and expected number of cases within the possible cluster^90^. The Gini index was used in the univariate and multivariate purely spatial analyses were used to determine the maximum population size of the scan windows. This Gini index was used to optimize the size of the population included in a specific cluster, to avoid finding the big ones only^91^. The same value obtained for purely spatial analysis was considered in space-time and spatial variation in the temporal trend analyses. The month range was used as the aggregation time for purely temporal, seasonal, and space-time analyses, whereas the year range was used for spatial variation analysis in temporal trends.. In this last analysis, the scan statistics estimate the annual percentage increase (or decrease) of temporal trends inside and outside the possible clusters. It identifies clusters where the inside temporal trends are statistically different from the outer ones^90^. In addition, multivariate analyses (purely spatial and space-time) revealed only simultaneous clusters for chikungunya and Zika, with RR >=1 for both diseases. High-risk clusters with *p* values $<5\%$ were considered significant. Subsequently, SatScan results were imported into QGIS software version 3.16^85^ to create thematic maps.

The values of environmental (temperatures, precipitation, and NDVI) and socioeconomic (BDI, sewage, rainwater system, and septic tank) variables of the sets of municipalities considered to be at high-risk in purely spatial univariate analyses for both diseases were statistically compared, with the respective values for municipalities with no risk (not included in the high-risk clusters) using a t-test between means. The normality of distributions and homogeneity of variances were evaluated using the Shapiro-Wilk, Anderson-Darling, and Levene tests. The comparison between the means of the groups and the tests was performed using the rstatix and car packages in the R software^86^. The two-sample Welch’s t-test for independent samples was used for normally distributed data with non-homogeneous variance. Statistical significance was set at *p* value $<5\%$.

The data obtained from Worldclim Version 2.1^92^ on maximum and minimum temperatures and mean precipitation for each month between 2015 and 2021, at 2.5 min resolution ( 21 $\hbox {km}^2$)^93^, were statistically analyzed using t-test. The rasters obtained were used to calculate the mean of the summers (considering January, February, and March) and the annual mean for 2015–2021 for the three climatic variables described above and for each municipality in the present study. These means were obtained by considering the climatic variable values contained in the pixels within each Brazilian municipality. Notably, these means were weighted based on the size of the area ($\hbox {km}^2$) of each pixel to deal with those located at the borders, which were only partially contained in a given municipality. Geographic operations (transformation in plane coordinates [Albers South America, ESRI:102033], raster vectorization, cutting, intersection, and dissolution of layers) from QGIS software version 3.16^85^ and ArcGis Pro software version 2.8^94^ were used.

Vegetation information was also considered using the NDVI values for each year from 2015 to 2021. The chosen source was the Terra and Aqua Moderate Resolution Imaging Spectroradiometer (MODIS) Vegetation Indices Combined 16-Day NDVI (MCD43A4) Version 6.1 satellite^95^, with a resolution of 463 m present on the Google Earth Engine (GEE) platform. Because Zika and chikungunya occur mostly in urban areas, NDVI values were obtained for the urban areas of the municipalities acquired from IBGE^96^. NDVI pixel means were calculated for these areas using Python language and geemap^97^ and eemont^98^ packages.Then, the average of 2015–2021 was calculated for the urban area of each municipality, and these values were used in the t-test.

BDI data calculated from income, education, and living condition indicators of the population in each municipality were used, considering the 2010 IBGE census. The data were obtained from Cidacs/Fiocruz Bahia^74^ (https://cidacs.bahia.fiocruz.br/ibp/). The percentage of permanent private households per municipality was also used according to the type of sewage system (sewage, rainwater system, and septic tank), type of piped water supply, and destination of the garbage collected by cleaning services. This information was obtained from IBGE^99^.These data were statistically analyzed using t-test.

**Table 1:** Table with previous studies considered cluster analysis of the co-occurrence of Zika and chikungunya analyzed simultaneously.

Methods	Variables	Events	Location	Period	Main findings	References
Scan statistics multivariate	Dengue incidence CHIK incidence	dengue CHIKV	Colombia	2015–2016	Seasonal factors may influence their co-occurrence; clusters are a consequence of precipitation, temperature, and elevation ranges.	Desjardins et al.^39^
Scan statistics multivariate	Dengue incidence CHIK incidence ZIKV incidence	dengue CHIKV ZIKV	Colombia	2014–2018	35% of cluster simultaneous for tree arboviruses; 2% dengue e CHIKV; 10% for dengue and ZIKV.	Freitas et al.^40^
Bayesian hierarchical Poisson	Dengue incidence ZIKV incidence	dengue ZIKV	Colombia	2015–2016	dengue high-risk associated with Zika high-risk.	Martínez- Bello et al.^34^
Gi* local spatial Kendall W test	Dengue incidence CHIK incidence ZIKV incidence	dengue CHIKV ZIKV	Merida Mexico,	2008–2015	The three viruses had significant agreement in their spatio-temporal distribution	Bisanzio et al^26^
Scan statistics multivariate	Dengue incidence CHIK incidence ZIKV incidence	dengue CHIKV ZIKV	Rio de Janeiro, Brazil	2015–2016	56% of cluster simultaneously for tree arboviruses, 16% for dengue and zika. Simultaneous clusters were found in areas of high population density, low socioeconomic status, rainy and warm seasons.	Freitas et al.^41^
Bayesian paradigm; multivariate Poisson	Dengue incidence CHIK incidence ZIKV incidence Social development index (SDI) green area pop. dens.	dengue CHIKV ZIKV	Rio de Janeiro, Brazil	2015–2016	Chikungunya was associated with a smaller 16% for dengue and zika. proportion of green area in comparison to dengue and Zika.	Schmidt et al.^42^
Bayesian hierarchical local empirical Bayesian bivariate global Moran	All incidence* Dengue incidence CHIK incidence ZIKV incidence SDI, water, income, garbage, sewage, pop. dens., Urban	dengue CHIKV ZIKV	Rio de Janeiro, Brazil	2015–2016	Tree arboviruses have a negative relationship between mean income. Zika had associated with less sewage. Chikungunya had associated a more urban area.	Queiroz and Medronho et al.^43^
Scan statistics multivariate	Dengue incidence CHIK incidence ZIKV incidence SDI	dengue CHIKV ZIKV	Rio de Janeiro, Brazil	2018	Over-risk for arboviruses in areas with the worst socioeconomic conditions.	Souza- Santos et al.^44^
multiple multilevel logistic regression	All incidence* socio- demographic income	dengue CHIKV ZIKV	Mangui- nhos, Rio de Janeiro, Brazil	2015–2016	High-rick of Arbovirus infection in areas with poor neighborhoods.	Rodrigues et al.^45^
Bayesian inference, INLA	Dengue incidence CHIK incidence temperature, humidity, rain, water, garbage, sewage, ruralility, income, illiterate	dengue CHIKV	Ceará, Brazil	2016–2021	7% of cluster simultaneously, which suggests a competition between viruses.	Pavani et al.^46^
Scan statistic univariate	All incidence* microcephaly incidence	dengue CHIKV ZIKV Microce- phaly	Pernambuco, Brazil	2014–2017	Vulnerable areas to underreporting were identified, comparing high risk clusters of microcephaly overlapping with low-risk clusters of diseases transmitted by *Aedes*.	Freitas et al.^47^
Bayesian hierarchical Poisson INLA	All incidence* Dengue incidence CHIK incidence ZIKV incidence education, overcrowding, water, sanitation health care centers	dengue CHIKV ZIKV	Medellin, Colombia; Fortaleza, Brazil	2014–2017	Dengue had an association with low socioeconomic status. Chikungunya had association with nonmonotonic socioeconomic measures. Zika has association few if any inequalities.	Carabali et al.^30^
Univariate and bivariate spatial, global and local Moran index	All incidence* Dengue incidence CHIK incidence ZIKV incidence pop. dens.,Gini	dengue CHIKV ZIKV	Maranhão, Brazil	2015–2016	autocorrelation of incidence rates of dengue and zika. socio-demographic factors influenced the occurrence of three diseases.	Costa et al.^27^

*All incidence = Incidence of the sum of three diseases cases: dengue, CHIKV and ZIKV.

**Table 2:** Number of confirmed cases and epidemiological profile of chikungunya and Zika in Brazil, with symptoms onset between 2015 and 2021.

	Chikungunya		Zika	
	Case	%	Case	%
**Criterion**				
Laboratory: PCR	11197	1.79		
Laboratory: Serological	126568	20.22		
Laboratory: Viral Isolation	990	0.16		
Laboratory: Other Methods	55983	8.94	18927	11.38
Clinical-epidemiological	431266	68.89	147390	88.62
**Classification**				
Autochthonous	381571	60.95	126821	76.25
Imported	17110	2.73	3038	1.83
Undetermined	41446	6.62	36432	21.91
Opened	185877	29.69	26	0.02
**Sex**				
Female	389460	62.21	111868	67.26
Masculine	236032	37.71	54161	32.57
Ignored	512	0.08	288	0.17
**Race/color**				
White	87537	13.98	34560	20.78
Black/Pardo	336318	53.73	61785	37.15
Others	7972	1.27	1417	0.85
Ignored	194177	31.02	68555	41.22
**Education**				
Low	89085	14.23	21434	12.89
Average	81225	12.98	24408	14.68
High	22864	3.65	8663	5.21
Not applicable	27406	4.38	11820	7.11
Ignored	405424	64.76	99992	60.12
**Hospitalization**				
Yes	11263	1.80		
Not	296876	47.42		
Ignored	317865	50.78		
**Clinic**				
Acute	438358	70.03		
Chronic	8414	1.34		
Ignored	179232	28.63		
**Total**	**626.004**	**100**	**166317**	**100**

**Table 3:** Statistical comparison of the means (t-test) of environmental and socioeconomic variables of municipalities included in the highest risk cluster with those not included in the purely spatial clusters of chikungunya and Zika in Brazil between 2015 and 2021.

	Chikungunya			Zika		
Variable	High risk cluster	No cluster	t	High risk cluster	No cluster	t
(Average)	(N=707)	(N=4863)		(N=520)	(N=5050)	
**Climate (2015–2021)**						
**Temperature (C°):**						
Maximum	29.80 (1.96)	28.44 (2.96)	15.997*	30.70 (1.90)	28.40(2,89)	24.790*
Maximum in summer	30.82(1.22)	29.56 (1.73)	24.109*	30.42(1.09)	26.65(1.76)	14.282*
Minimum	19.81(1.94)	17.21 (3.14)	29.675*	18.77 (1.71)	17.41(3.22)	15.587*
Minimum in summer	20.94(1.55)	19.31 (2.01)	24.939*	20.21 (1.32)	19.44(2.14)	11.875*
**Precipitation (mm):**						
Average	80.65 (31.21)	111.98 (36.80)	$-24.335$*	116.18(32.28)	107.16(38.02)	5.962**
Average in summer	120.29(70.20)	178.66 (64.98)	$-20.847$*	217.19 (63.10)	166.52(66.95)	16.626*
**Environmental (2015–2021)**						
NDVI in urban areas	0.49 (0.09)	0.52 (0.10)	− 10.455*	0.51 (0.08)	0.52 (0.10)	− 4.159*
**Socio-economic (2010)**						
BDI	0.73 (0.79)	− 0.11 (0.98)	25.439*	− 0.11 (0.72)	0.01(1.02)	− 3.497*
**% of households:**						
Piped water	67.22(18.53)	69.45(20.00)	− 2.963**	68.85(17.19)	69.20(20.08)	NS
Sewerage system	33.33(25.84)	43.62(31.78)	− 9.587*	28.96(28.14)	43.69(31.26)	− 11.240*
Garbage collected	52.43(24.69)	63.16(26.20)	− 10.712*	65.22(22.26)	61.45(26.61)	3.607*

*p value $<0.001$. **p value $<0.01$. *NS* Not significant. Numbers in parentheses indicate the standard deviation.

### Ethical approval

The study was approved by the School of Public Health - University of São Paulo, Committee for Ethics in Research (COEP), the Plataforma Brasil system, Ministry of Health, number CAAE: 55947522.0.0000.5421.

### Supplementary Information

Supplementary Figures.

Supplementary Tables.

Supplementary Tables.

## Data Availability

Secondary data for chikungunya and Zika in Brazil, 2015-2021, obtained by consulting the Ministério da Saúde - DATASUS repository^84^ in 13th May 2022. This data are available in https://datasus.saude.gov.br/transferencia-de-arquivos/. The datasets and data analysed generated during the current study are included in this published article, Supplementary Tables S1 and S7.
