# Supplementary material for: Zika, chikungunya and co-occurrence in Brazil: space-time clusters and associated environmental–socioeconomic factors
Source: Sci Rep. 2023 Oct 21;13:18026. doi: 10.1038/s41598-023-42930-4 (PMC10590386; doi:10.1038/s41598-023-42930-4)
Supplement: Supplementary file 1 — Supplementary Figures. [file 41598_2023_42930_MOESM1_ESM.docx]

**Supplementary appendix**

**
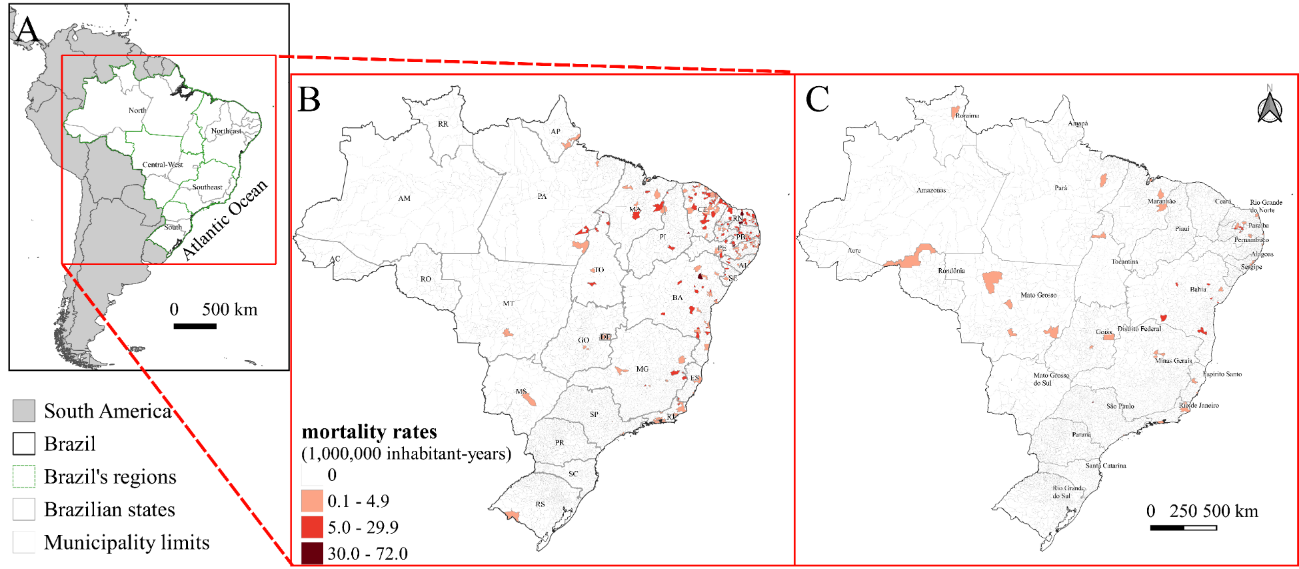
Fig. S1** – Map of South America, Brazil, and its regions (**a**), map of Brazil and its states, with the mortality rate (100,000 inhabitants-year) of confirmed chikungunya (**b**) and Zika (**c**) deaths based on the municipality of residence, with symptom onset between 2015 and 2021. (**b**) Legend with the abbreviations of the states, (**c**) legend with the names of the states.

**
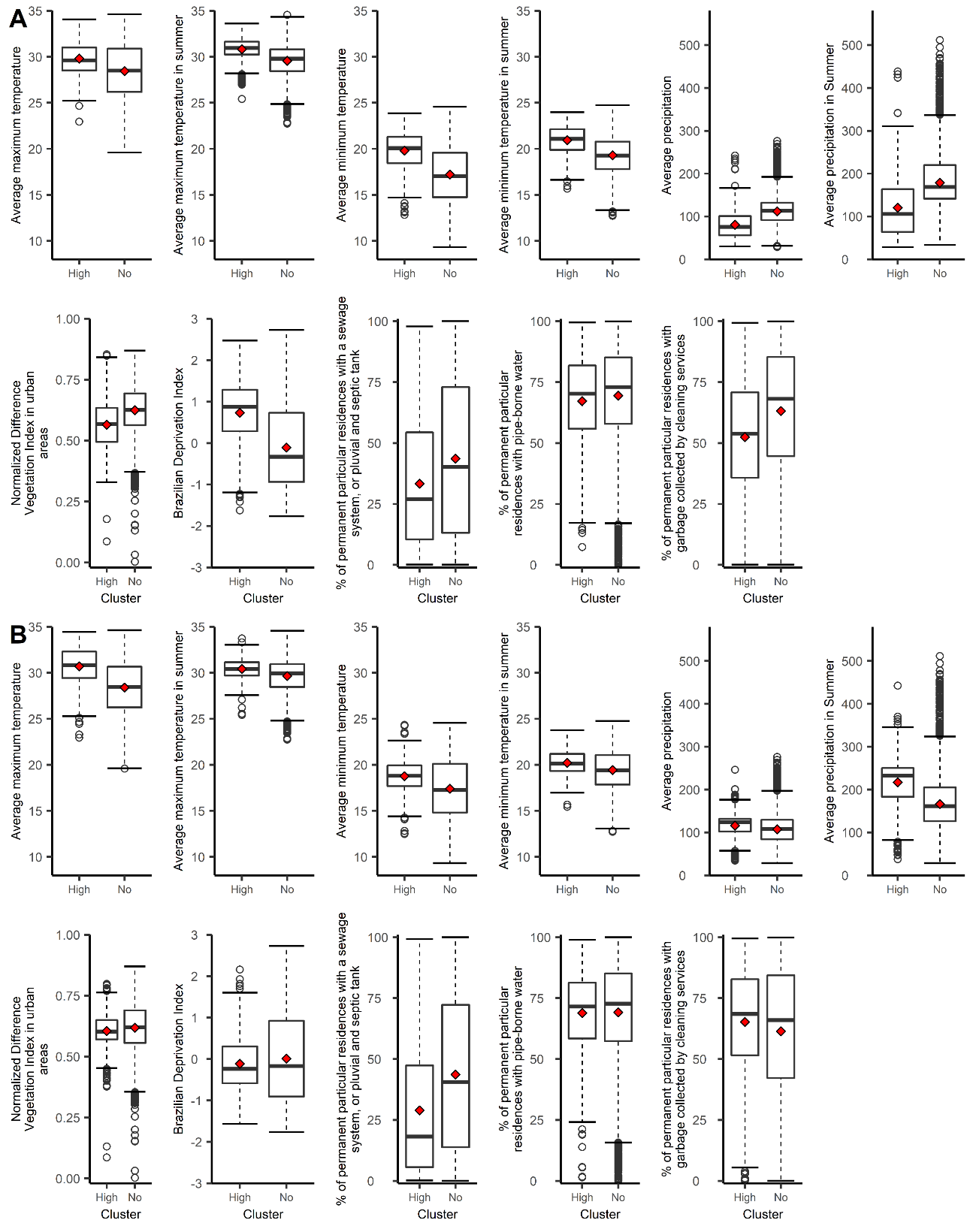
**

**Fig. S2** – Boxplot of climatic, environmental (average of 2015-2021) and socioeconomic variables (2010) of the municipalities of residence included in the clusters of high-risk chikungunya (**a**) and Zika (**b**) cases and those of Brazilian municipalities not included in the purely spatial clusters, for the period 2015-2021. Point in red average of the group.


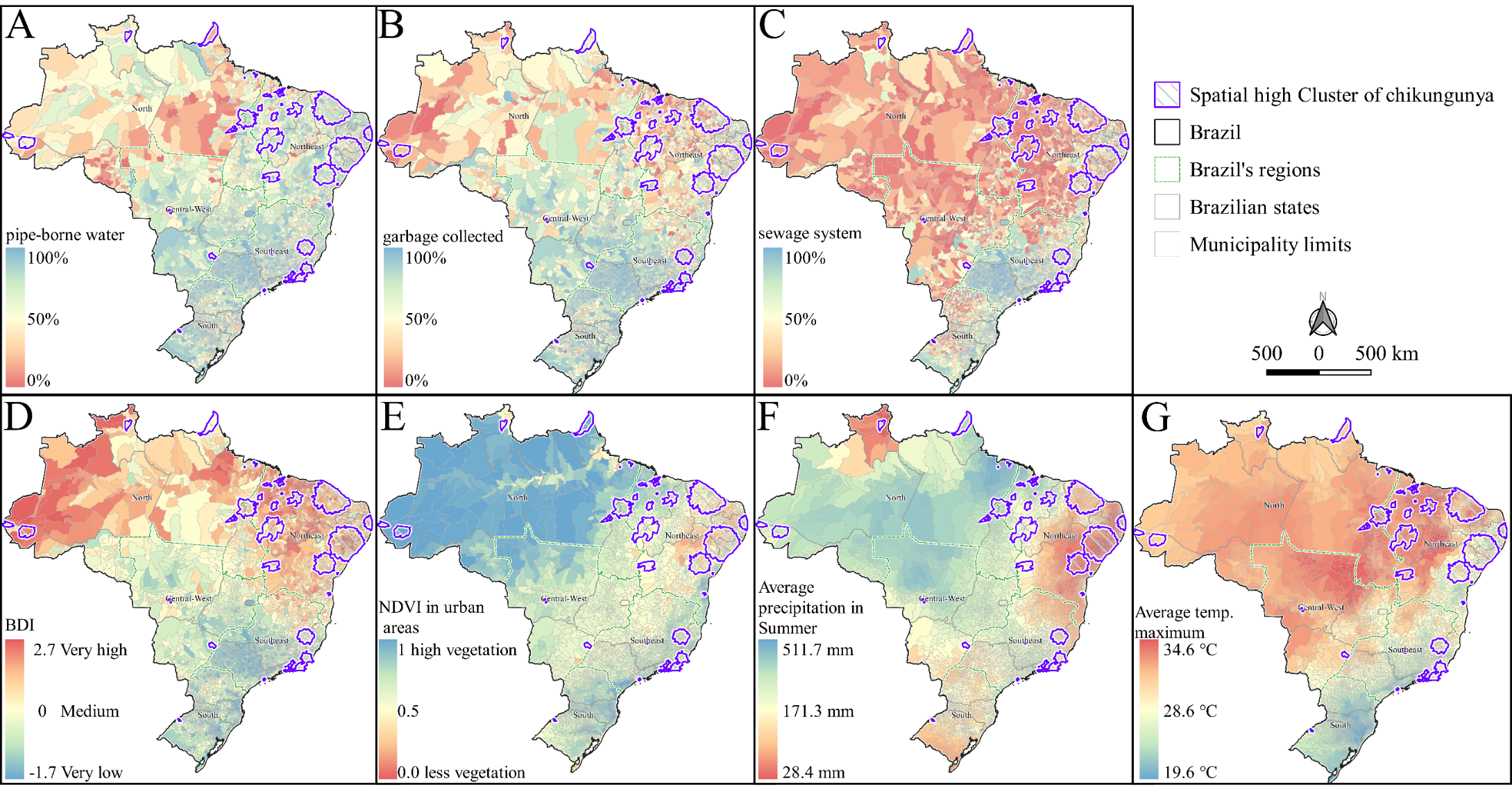


**Fig. S3 –** Map of socioeconomic variables to 2010 (% of households: **a**-piped water, **b**-garbage collected, **c**-sewerage and septic tank and **d**-BDI) and environmental variables average of 2015-2021 (**e**-NDVI in urban area, **f**-precipitation, **g**-temperature) superimposed on the municipalities included in the highest risk cluster in the purely spatial analysis of chikungunya cases in Brazil from 2015 to 2021.


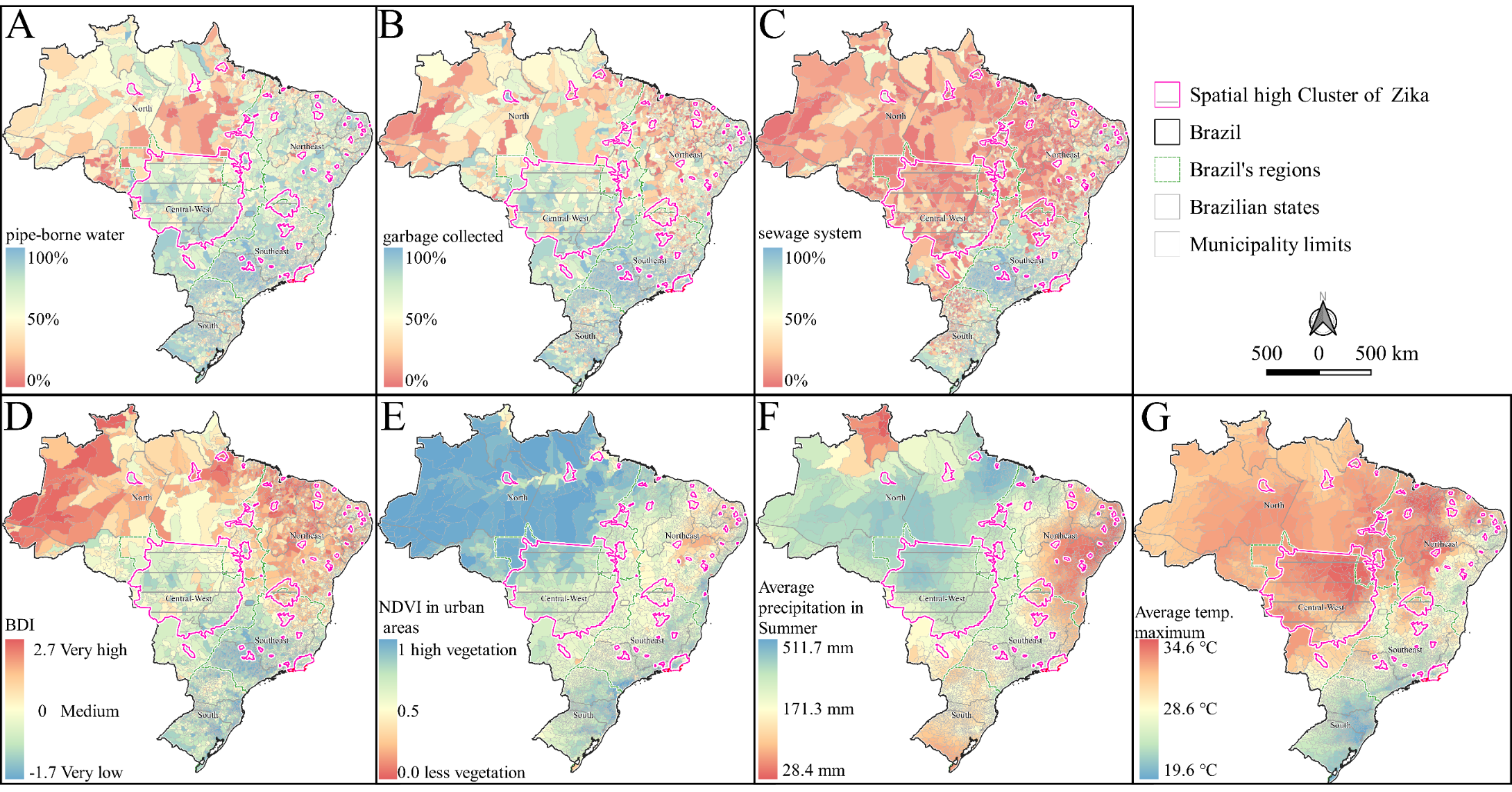


**Fig. S4 –** Map of socioeconomic variables to 2010 (% of households: **a**-piped water, **b**-garbage collected, **c**-sewerage and septic tank and **d**-BDI) and environmental variables average of 2015-2021 (**e**-NDVI in urban area, **f**-precipitation, **g**-temperature) superimposed on the municipalities included in the highest risk cluster in the purely spatial analysis of Zika cases in Brazil from 2015 to 2021.
