# Supplementary material for: Zika, chikungunya and co-occurrence in Brazil: space-time clusters and associated environmental–socioeconomic factors
Source: Sci Rep. 2023 Oct 21;13:18026. doi: 10.1038/s41598-023-42930-4 (PMC10590386; doi:10.1038/s41598-023-42930-4)
Supplement: Supplementary file 3 — Supplementary Tables. [file 41598_2023_42930_MOESM3_ESM.docx]

**Supplementary appendix**

**Table S2.** Incidence and mortality rates (1,000,000 inhabitant-years) and number of chikungunya and Zika cases per municipality, estates, and region at Brazil, 2015-2021. MN= number of municipality. *6 and 1 cases of chikungunya and Zika, respectively, no data on the municipality of residence.

| **chikungunya** | | | | | | | | | | | | | | | | | | | | | | | | | | | |
| --- | --- | --- | --- | --- | --- | --- | --- | --- | --- | --- | --- | --- | --- | --- | --- | --- | --- | --- | --- | --- | --- | --- | --- | --- | --- | --- | --- |
| **Region** | **UF** | **MN** |  |  | **Incidence rates** | | | | **No. cases** | | | | |  | |  | | **Mortality rates** | | | | **No. death** | | | | | |
|  |  | **total** | **MN** | **(%)** | **min** | | **max** | | **n*** | | **min max** | | | **MN** | | **(%)** | | **min** | | **max** | | **n** | | | **min max** | | |
| **Center-west** | **DF** | 1 | 1 | 100.0 | 2.22 | | - | | 462 | | - | | - | 1 | | 100.0 | | 0.10 | | - | | 2 | | | - | - | |
|  | **GO** | 246 | 71 | 28.9 | 0.07 | | 91.69 | | 544 | | 1 | | 131 | 2 | | 0.8 | | 0.25 | | 1.14 | | 2 | | | 1 | 1 | |
|  | **MS** | 79 | 52 | 65.8 | 0.43 | | 98.67 | | 672 | | 1 | | 235 | 1 | | 1.3 | | 0.16 | | - | | 1 | | | - | - | |
|  | **MT** | 141 | 90 | 63.8 | 0.38 | | 629.38 | | 17087 | | 1 | | 12409 | 2 | | 1.4 | | 1.52 | | 1.65 | | 10 | | | 3 | 7 | |
| **Northeast** | **AL** | 102 | 94 | 92.2 | 0.94 | | 326.04 | | 15876 | | 1 | | 6306 | 5 | | 4.9 | | 0.62 | | 15.32 | | 16 | | | 1 | 11 | |
|  | **BA** | 417 | 338 | 81.1 | 0.29 | | 1162.70 | | 81180 | | 1 | | 14551 | 23 | | 5.5 | | 0.25 | | 35.65 | | 44 | | | 1 | 9 | |
|  | **CE** | 184 | 180 | 97.8 | 0.35 | | 791.05 | | 142818 | | 1 | | 81089 | 42 | | 22.8 | | 1.05 | | 19.00 | | 247 | | | 1 | 172 | |
|  | **MA** | 217 | 175 | 80.6 | 0.31 | | 620.79 | | 16369 | | 1 | | 4169 | 9 | | 4.1 | | 0.85 | | 6.53 | | 33 | | | 1 | 16 | |
|  | **PB** | 223 | 172 | 77.1 | 0.93 | | 863.27 | | 22910 | | 1 | | 9310 | 27 | | 12.1 | | 1.75 | | 41.22 | | 57 | | | 1 | 23 | |
|  | **PE** | 185 | 178 | 96.2 | 0.34 | | 841.73 | | 60397 | | 1 | | 27274 | 31 | | 16.8 | | 0.61 | | 17.41 | | 129 | | | 1 | 70 | |
|  | **PI** | 224 | 108 | 48.2 | 0.36 | | 343.62 | | 8606 | | 1 | | 5130 | 4 | | 1.8 | | 0.83 | | 44.97 | | 9 | | | 1 | 5 | |
|  | **RN** | 167 | 148 | 88.6 | 0.93 | | 642.40 | | 20580 | | 1 | | 7907 | 31 | | 18.6 | | 2.23 | | 72.04 | | 129 | | | 1 | 49 | |
|  | **SE** | 75 | 71 | 94.7 | 0.61 | | 622.12 | | 16244 | | 1 | | 4568 | 6 | | 8.0 | | 0.66 | | 21.90 | | 8 | | | 1 | 3 | |
| **North** | **AC** | 22 | 20 | 90.9 | 0.32 | | 72.82 | | 745 | | 1 | | 238 |  | |  | |  | |  | |  | | |  |  | |
|  | **AM** | 62 | 25 | 40.3 | 0.29 | | 11.27 | | 515 | | 1 | | 352 |  | |  | |  | |  | |  | | |  |  | |
|  | **AP** | 16 | 13 | 81.3 | 0.85 | | 498.96 | | 1683 | | 1 | | 930 | 1 | | 6.3 | | 0.29 | | - | | 1 | | | - | - | |
|  | **PA** | 144 | 117 | 81.3 | 0.25 | | 487.34 | | 17853 | | 1 | | 5938 | 4 | | 2.8 | | 0.10 | | 16.08 | | 8 | | | 1 | 5 | |
|  | **RO** | 52 | 38 | 73.1 | 0.59 | | 91.28 | | 477 | | 1 | | 185 |  | |  | |  | |  | |  | | |  |  | |
|  | **RR** | 15 | 15 | 100.0 | 1.29 | | 144.69 | | 4102 | | 1 | | 3802 |  | |  | |  | |  | |  | | |  |  | |
|  | **TO** | 139 | 79 | 56.8 | 1.10 | | 203.40 | | 3188 | | 1 | | 744 | 5 | | 3.6 | | 2.82 | | 35.20 | | 5 | | | 1 | 1 | |
| **Southeast** | **ES** | 78 | 65 | 83.3 | 0.49 | | 212.09 | | 7555 | | 1 | | 5319 | 4 | | 5.1 | | 0.40 | | 13.52 | | 5 | | | 1 | 2 | |
|  | **MG** | 853 | 372 | 43.6 | 0.11 | | 1128.38 | | 33917 | | 1 | | 10763 | 9 | | 1.1 | | 0.22 | | 20.36 | | 21 | | | 1 | 12 | |
|  | **RJ** | 92 | 89 | 96.7 | 0.52 | | 627.28 | | 132897 | | 1 | | 65988 | 17 | | 18.5 | | 0.40 | | 18.91 | | 114 | | | 1 | 77 | |
|  | **SP** | 645 | 297 | 46.0 | 0.10 | | 253.21 | | 18115 | | 1 | | 7674 | 4 | | 0.6 | | 0.63 | | 1.18 | | 8 | | | 1 | 3 | |
| **South** | **PR** | 399 | 95 | 23.8 | 0.12 | | 22.38 | | 468 | | 1 | | 93 |  | |  | |  | |  | |  | | |  |  | |
|  | **RS** | 497 | 70 | 14.1 | 0.04 | | 630.45 | | 438 | | 1 | | 235 | 1 | | 0.2 | | 1.84 | | - | | 1 | | | - | - | |
|  | **SC** | 295 | 69 | 23.4 | 0.07 | | 12.21 | | 300 | | 1 | | 48 |  | |  | |  | |  | |  | | |  |  | |
| **Zika** | | | | | | | | | | | | | | | | | | | | | | | | | | | |
|  |  | **MN** |  |  | **Incidence rates** | | | **No. cases** | | | | | | |  | |  | | **Mortality rates** | | | | **No. death** | | | | |
| **Region** | **UF** | **total** | **MN** | **(%)** | **min** | **max** | | **n*** | | **min max** | | | | | **MN** | | **(%)** | | **min** | | **max** | | **n** | **min max** | | | |
| **Center-west** | **DF** | 1 | 1 | 100.0 | 1.42 | - | | 296 | | - | | - | | | 1 | | 100.0 | | 0.05 | | - | | 1 | - | | | - |
|  | **GO** | 246 | 101 | 41.1 | 0.12 | 151.54 | | 9831 | | 1 | | 7211 | | | 1 | | 0.4 | | 0.19 | | - | | 2 | - | | | - |
|  | **MS** | 79 | 38 | 48.1 | 0.26 | 121.45 | | 2038 | | 1 | | 1582 | | |  | |  | |  | |  | |  |  | | |  |
|  | **MT** | 141 | 115 | 81.6 | 0.84 | 601.67 | | 18958 | | 1 | | 4582 | | | 4 | | 2.8 | | 1.65 | | 4.71 | | 11 | 1 | | | 7 |
| **Northeast** | **AL** | 102 | 82 | 80.4 | 0.57 | 193.56 | | 4937 | | 1 | | 2151 | | | 3 | | 2.9 | | 0.14 | | 9.05 | | 3 | 1 | | | 1 |
|  | **BA** | 417 | 252 | 60.4 | 0.22 | 1198.24 | | 27486 | | 1 | | 17844 | | | 9 | | 2.2 | | 3.62 | | 14.31 | | 12 | 1 | | | 3 |
|  | **CE** | 184 | 90 | 48.9 | 0.11 | 148.35 | | 3369 | | 1 | | 1642 | | |  | |  | |  | |  | |  |  | | |  |
|  | **MA** | 217 | 91 | 41.9 | 0.21 | 94.61 | | 4648 | | 1 | | 2935 | | | 3 | | 1.4 | | 0.52 | | 1.81 | | 6 | 1 | | | 4 |
|  | **PB** | 223 | 114 | 51.1 | 0.50 | 149.56 | | 2988 | | 1 | | 606 | | | 4 | | 1.8 | | 0.35 | | 20.11 | | 8 | 1 | | | 5 |
|  | **PE** | 185 | 77 | 41.6 | 0.14 | 41.08 | | 465 | | 1 | | 177 | | | 1 | | 0.5 | | 0.09 | | - | | 1 | - | | | - |
|  | **PI** | 224 | 43 | 19.2 | 0.32 | 22.94 | | 218 | | 1 | | 62 | | |  | |  | |  | |  | |  |  | | |  |
|  | **RN** | 167 | 72 | 43.1 | 0.52 | 111.53 | | 900 | | 1 | | 222 | | | 7 | | 4.2 | | 0.49 | | 23.12 | | 9 | 1 | | | 3 |
|  | **SE** | 75 | 34 | 45.3 | 0.28 | 41.30 | | 593 | | 1 | | 203 | | |  | |  | |  | |  | |  |  | | |  |
| **North** | **AC** | 22 | 11 | 50.0 | 0.78 | 90.45 | | 521 | | 1 | | 146 | | |  | |  | |  | |  | |  |  | | |  |
|  | **AM** | 62 | 25 | 40.3 | 0.22 | 33.25 | | 5152 | | 1 | | 4993 | | |  | |  | |  | |  | |  |  | | |  |
|  | **AP** | 16 | 7 | 43.8 | 1.43 | 17.17 | | 474 | | 1 | | 412 | | |  | |  | |  | |  | |  |  | | |  |
|  | **PA** | 144 | 79 | 54.9 | 0.13 | 211.48 | | 3704 | | 1 | | 2138 | | | 1 | | 0.7 | | 2.27 | | - | | 1 | - | | | - |
|  | **RO** | 52 | 29 | 55.8 | 0.37 | 48.08 | | 859 | | 1 | | 328 | | | 1 | | 1.9 | | 0.28 | | - | | 1 | - | | | - |
|  | **RR** | 15 | 10 | 66.7 | 1.23 | 28.39 | | 426 | | 1 | | 362 | | | 1 | | 6.7 | | 0.76 | | - | | 2 | - | | | - |
|  | **TO** | 139 | 70 | 50.4 | 1.19 | 140.90 | | 2525 | | 1 | | 1110 | | | 1 | | 0.7 | | 0.81 | | - | | 1 | - | | | - |
| **Southeast** | **ES** | 78 | 51 | 65.4 | 0.08 | 58.34 | | 3430 | | 1 | | 1463 | | | 2 | | 2.6 | | 0.29 | | 1.18 | | 2 | 1 | | | 1 |
|  | **MG** | 853 | 238 | 27.9 | 0.16 | 220.22 | | 10607 | | 1 | | 2567 | | | 1 | | 0.1 | | 2.88 | | - | | 1 | - | | | - |
|  | **RJ** | 92 | 73 | 79.3 | 0.32 | 277.91 | | 56335 | | 1 | | 35090 | | | 2 | | 2.2 | | 0.11 | | 1.70 | | 11 | 5 | | | 6 |
|  | **SP** | 645 | 180 | 27.9 | 0.02 | 165.46 | | 4907 | | 1 | | 1042 | | | 2 | | 0.3 | | 0.45 | | 47.68 | | 2 | 1 | | | 1 |
| **South** | **PR** | 399 | 94 | 23.6 | 0.04 | 53.25 | | 415 | | 1 | | 45 | | |  | |  | |  | |  | |  |  | | |  |
|  | **RS** | 497 | 47 | 9.5 | 0.03 | 10.73 | | 183 | | 1 | | 44 | | |  | |  | |  | |  | |  |  | | |  |
|  | **SC** | 295 | 26 | 8.8 | 0.02 | 8.68 | | 51 | | 1 | | 15 | | |  | |  | |  | |  | |  |  | | |  |

**Table S3.** Spatial analysis cluster of chikungunya and Zika cases at Brazil, 2015-2021.

*C = clusters’ identification number, see Fig. 4b and 4b. Nº M = Number of municipalities.

| **chikungunya** | | | | | | **Zika** | | | | | | |
| --- | --- | --- | --- | --- | --- | --- | --- | --- | --- | --- | --- | --- |
| **C*** | **Nº M** | **Observed** | **Expected** | **RR** | **p-value** | **C*** | **Nº M** | **Observed** | **Expected** | **RR** | **p-value** |  |
| 1 | 133 | 140442 | 21477.30 | 8.20 | 0.001 | 1 | 1 | 17816 | 169.28 | 118.04 | 0.001 |  |
| 2 | 17 | 25433 | 2672.58 | 9.89 | 0.001 | 2 | 1 | 34885 | 5185.45 | 8.29 | 0.001 |  |
| 3 | 60 | 28671 | 4036.86 | 7.40 | 0.001 | 3 | 299 | 27583 | 5581.06 | 5.75 | 0.001 |  |
| 4 | 2 | 14699 | 652.60 | 23.06 | 0.001 | 4 | 49 | 16161 | 3249.41 | 5.41 | 0.001 |  |
| 5 | 1 | 61730 | 20315.34 | 3.27 | 0.001 | 5 | 8 | 3946 | 357.08 | 11.30 | 0.001 |  |
| 6 | 73 | 52378 | 15281.90 | 3.66 | 0.001 | 6 | 1 | 1013 | 59.50 | 17.13 | 0.001 |  |
| 7 | 1 | 12376 | 801.52 | 15.74 | 0.001 | 7 | 1 | 4805 | 1721.30 | 2.85 | 0.001 |  |
| 8 | 55 | 23091 | 5401.67 | 4.40 | 0.001 | 8 | 1 | 589 | 10.56 | 56.00 | 0.001 |  |
| 9 | 244 | 44313 | 21435.66 | 2.15 | 0.001 | 9 | 2 | 3186 | 1036.16 | 3.12 | 0.001 |  |
| 10 | 2 | 11153 | 2280.88 | 4.96 | 0.001 | 10 | 1 | 475 | 22.21 | 21.44 | 0.001 |  |
| 11 | 2 | 4528 | 496.17 | 9.19 | 0.001 | 11 | 8 | 1352 | 312.77 | 4.35 | 0.001 |  |
| 12 | 19 | 19426 | 8770.41 | 2.26 | 0.001 | 12 | 1 | 754 | 94.16 | 8.04 | 0.001 |  |
| 13 | 1 | 2451 | 252.84 | 9.73 | 0.001 | 13 | 1 | 506 | 33.62 | 15.10 | 0.001 |  |
| 14 | 9 | 3017 | 498.84 | 6.07 | 0.001 | 14 | 43 | 1868 | 610.18 | 3.09 | 0.001 |  |
| 15 | 1 | 1513 | 122.85 | 12.34 | 0.001 | 15 | 1 | 458 | 34.47 | 13.32 | 0.001 |  |
| 16 | 1 | 2179 | 360.66 | 6.06 | 0.001 | 16 | 1 | 353 | 31.26 | 11.31 | 0.001 |  |
| 17 | 1 | 3726 | 1035.03 | 3.62 | 0.001 | 17 | 7 | 550 | 90.77 | 6.08 | 0.001 |  |
| 18 | 1 | 926 | 68.43 | 13.55 | 0.001 | 18 | 1 | 1575 | 696.28 | 2.27 | 0.001 |  |
| 19 | 1 | 980 | 130.46 | 7.52 | 0.001 | 19 | 1 | 324 | 40.29 | 8.06 | 0.001 |  |
| 20 | 10 | 6765 | 3665.71 | 1.85 | 0.001 | 20 | 1 | 370 | 58.07 | 6.38 | 0.001 |  |
| 21 | 41 | 3870 | 1931.65 | 2.01 | 0.001 | 21 | 1 | 211 | 14.16 | 14.92 | 0.001 |  |
| 22 | 1 | 1463 | 433.73 | 3.38 | 0.001 | 22 | 1 | 2122 | 1192.12 | 1.79 | 0.001 |  |
| 23 | 5 | 1376 | 419.90 | 3.28 | 0.001 | 23 | 1 | 398 | 88.28 | 4.52 | 0.001 |  |
| 24 | 1 | 235 | 16.25 | 14.47 | 0.001 | 24 | 2 | 190 | 17.43 | 10.91 | 0.001 |  |
| 25 | 1 | 435 | 74.46 | 5.85 | 0.001 | 25 | 1 | 214 | 24.86 | 8.62 | 0.001 |  |
| 26 | 1 | 807 | 344.50 | 2.34 | 0.001 | 26 | 20 | 686 | 243.67 | 2.82 | 0.001 |  |
| 27 | 1 | 557 | 241.57 | 2.31 | 0.001 | 27 | 1 | 163 | 14.23 | 11.47 | 0.001 |  |
| 28 | 5 | 3402 | 2731.43 | 1.25 | 0.001 | 28 | 1 | 138 | 9.73 | 14.19 | 0.001 |  |
| 29 | 1 | 215 | 86.31 | 2.49 | 0.001 | 29 | 1 | 229 | 43.94 | 5.22 | 0.001 |  |
| 30 | 1 | 291 | 152.34 | 1.91 | 0.001 | 30 | 2 | 622 | 264.14 | 2.36 | 0.001 |  |
| 31 | 1 | 68 | 18.01 | 3.78 | 0.001 | 31 | 1 | 203 | 41.95 | 4.84 | 0.001 |  |
| 32 | 1 | 148 | 74.13 | 2.00 | 0.001 | 32 | 3 | 307 | 99.94 | 3.08 | 0.001 |  |
| 33 | 1 | 229 | 141.50 | 1.62 | 0.001 | 33 | 3 | 115 | 14.75 | 7.80 | 0.001 |  |
| 34 | 1 | 94 | 47.98 | 1.96 | 0.001 | 34 | 10 | 1136 | 676.07 | 1.69 | 0.001 |  |
| 35 | 8 | 3958 | 3607.27 | 1.10 | 0.001 | 35 | 1 | 94 | 10.94 | 8.60 | 0.001 |  |
| 36 | 1 | 164 | 103.55 | 1.58 | 0.002 | 36 | 1 | 578 | 285.94 | 2.03 | 0.001 |  |
| 37 | 1 | 276 | 195.69 | 1.41 | 0.003 | 37 | 1 | 217 | 64.28 | 3.38 | 0.001 |  |
| 38 | 1 | 45 | 18.99 | 2.37 | 0.016 | 38 | 7 | 229 | 80.30 | 2.85 | 0.001 |  |
|  |  |  |  |  |  | 39 | 1 | 113 | 23.68 | 4.78 | 0.001 |  |
|  |  |  |  |  |  | 40 | 4 | 125 | 33.18 | 3.77 | 0.001 |  |
|  |  |  |  |  |  | 41 | 1 | 190 | 67.74 | 2.81 | 0.001 |  |
|  |  |  |  |  |  | 42 | 6 | 183 | 64.89 | 2.82 | 0.001 |  |
|  |  |  |  |  |  | 43 | 1 | 112 | 29.32 | 3.82 | 0.001 |  |
|  |  |  |  |  |  | 44 | 7 | 180 | 68.09 | 2.65 | 0.001 |  |
|  |  |  |  |  |  | 45 | 1 | 89 | 22.65 | 3.93 | 0.001 |  |
|  |  |  |  |  |  | 46 | 5 | 178 | 83.56 | 2.13 | 0.001 |  |
|  |  |  |  |  |  | 47 | 1 | 23 | 1.80 | 12.79 | 0.001 |  |
|  |  |  |  |  |  | 48 | 1 | 81 | 27.29 | 2.97 | 0.001 |  |
|  |  |  |  |  |  | 49 | 1 | 307 | 187.90 | 1.64 | 0.001 |  |
|  |  |  |  |  |  | 50 | 1 | 44 | 11.21 | 3.93 | 0.001 |  |
|  |  |  |  |  |  | 51 | 1 | 17 | 2.32 | 7.32 | 0.001 |  |
|  |  |  |  |  |  | 52 | 1 | 60 | 24.44 | 2.46 | 0.002 |  |
|  |  |  |  |  |  | 53 | 1 | 26 | 7.14 | 3.64 | 0.004 |  |

**Table S4.** Spatial multivariate analysis cluster of chikungunya and Zika cases at Brazil, 2015-2021. *C = clusters’ identification number, see Fig. 4c. Nº M = Number of municipalities.

| **C*** | **Nº M** | **p-value** | **diseases** | **Observed** | **Expected** | **RR** |
| --- | --- | --- | --- | --- | --- | --- |
| 1 | 2 | 0.001 | chik | 14699 | 652.60 | 23.06 |
|  |  |  | Zika | 17904 | 173.67 | 115.69 |
| 2 | 1 | 0.001 | chik | 61730 | 20315.34 | 3.27 |
|  |  |  | Zika | 34885 | 5185.45 | 8.29 |
| 3 | 17 | 0.001 | chik | 25433 | 2672.58z | 9.89 |
|  |  |  | Zika | 5471 | 692.19 | 8.14 |
| 4 | 38 | 0.001 | chik | 25332 | 3112.78 | 8.45 |
|  |  |  | Zika | 5225 | 818.50 | 6.56 |
| 5 | 102 | 0.001 | chik | 16661 | 7624.95 | 2.22 |
|  |  |  | Zika | 17133 | 2102.67 | 8.99 |
| 6 | 1 | 0.001 | chik | 8533 | 3257.87 | 2.64 |
|  |  |  | Zika | 8451 | 848.06 | 10.46 |
| 7 | 2 | 0.001 | chik | 4528 | 496.17 | 9.19 |
|  |  |  | Zika | 303 | 140.46 | 2.16 |
| 8 | 1 | 0.001 | chik | 1513 | 122.85 | 12.34 |
|  |  |  | Zika | 73 | 35.12 | 2.08 |
| 9 | 1 | 0.001 | chik | 2179 | 360.66 | 6.06 |
|  |  |  | Zika | 207 | 104.58 | 1.98 |
| 10 | 1 | 0.001 | chik | 3726 | 1035.03 | 3.62 |
|  |  |  | Zika | 361 | 301.35 | 1.20 |
| 11 | 1 | 0.001 | chik | 926 | 68.43 | 13.55 |
|  |  |  | Zika | 31 | 20.79 | 1.49 |
| 12 | 2 | 0.001 | chik | 4430 | 3721.50 | 1.19 |
|  |  |  | Zika | 3186 | 1036.16 | 3.12 |
| 13 | 6 | 0.001 | chik | 3452 | 1425.11 | 2.43 |
|  |  |  | Zika | 395 | 380.61 | 1.04 |
| 14 | 5 | 0.001 | chik | 6015 | 4649.11 | 1.30 |
|  |  |  | Zika | 2123 | 1257.37 | 1.70 |
| 15 | 1 | 0.001 | chik | 229 | 141.50 | 1.62 |
|  |  |  | Zika | 324 | 40.29 | 8.06 |
| 16 | 1 | 0.001 | chik | 235 | 16.25 | 14.47 |
|  |  |  | Zika | 4 | 3.91 | 1.02 |
| 17 | 1 | 0.001 | chik | 557 | 241.57 | 2.31 |
|  |  |  | Zika | 190 | 67.74 | 2.81 |
| 18 | 1 | 0.001 | chik | 94 | 47.98 | 1.96 |
|  |  |  | Zika | 118 | 14.23 | 8.30 |
| 19 | 1 | 0.001 | chik | 164 | 103.55 | 1.58 |
|  |  |  | Zika | 118 | 31.36 | 3.77 |
| 20 | 1 | 0.008 | chik | 20 | 9.04 | 2.21 |
|  |  |  | Zika | 12 | 2.40 | 5.00 |

**Table S5.** Space-time analysis’ clusters of chikungunya and Zika cases at Brazil, 2015-2021. *Cluster = Clusters’ identification number, see Fig. 5a and 5b. Nº M = Number of municipalities.

|  | **C*** | **Start - End Date** | | **Duration (Month)** | | **Nº M** | | **Observed** | **Expected** | **RR** | **p-value** |
| --- | --- | --- | --- | --- | --- | --- | --- | --- | --- | --- | --- |
| **Chikungunya** | 1 | 2016/3/1 | 2017/7/31 | 17 | | 172 | | 133318 | 4651.13 | 36.43 | 0.001 |
|  | 2 | 2019/3/1 | 2019/7/31 | 5 | | 1 | | 31899 | 1201.02 | 27.97 | 0.001 |
|  | 3 | 2018/2/1 | 2019/8/31 | 19 | | 86 | | 44645 | 3927.27 | 12.19 | 0.001 |
|  | 4 | 2016/1/1 | 2016/5/31 | 5 | | 2 | | 13627 | 38.33 | 363.65 | 0.001 |
|  | 5 | 2017/2/1 | 2018/6/30 | 17 | | 38 | | 21340 | 619.43 | 35.67 | 0.001 |
|  | 6 | 2015/11/1 | 2016/7/31 | 9 | | 276 | | 31129 | 2520.19 | 12.96 | 0.001 |
|  | 7 | 2018/1/1 | 2018/4/30 | 4 | | 1 | | 9249 | 37.16 | 252.69 | 0.001 |
|  | 8 | 2021/4/1 | 2021/8/31 | 5 | | 136 | | 21803 | 1320.83 | 17.08 | 0.001 |
|  | 9 | 2021/2/1 | 2021/5/31 | 4 | | 7 | | 12911 | 231.17 | 57.04 | 0.001 |
|  | 10 | 2021/2/1 | 2021/5/31 | 4 | | 13 | | 4953 | 48.77 | 102.38 | 0.001 |
|  | 11 | 2016/2/1 | 2016/3/31 | 2 | | 1 | | 3161 | 4.29 | 740.43 | 0.001 |
|  | 12 | 2017/1/1 | 2017/5/31 | 5 | | 123 | | 8418 | 433.36 | 19.68 | 0.001 |
|  | 13 | 2019/3/1 | 2019/7/31 | 5 | | 22 | | 7385 | 423.91 | 17.62 | 0.001 |
|  | 14 | 2017/4/1 | 2017/8/31 | 5 | | 1 | | 3224 | 61.19 | 52.96 | 0.001 |
|  | 15 | 2016/3/1 | 2016/5/31 | 3 | | 87 | | 5823 | 472.09 | 12.44 | 0.001 |
|  | 16 | 2018/1/1 | 2019/3/31 | 15 | | 5 | | 6889 | 1172.93 | 5.93 | 0.001 |
|  | 17 | 2021/3/1 | 2021/4/30 | 2 | | 1 | | 212 | 0.38 | 553.85 | 0.001 |
|  | 18 | 2021/2/1 | 2021/5/31 | 4 | | 1 | | 267 | 7.06 | 37.81 | 0.001 |
|  | 19 | 2015/1/1 | 2015/2/28 | 2 | | 54 | | 667 | 148.40 | 4.50 | 0.001 |
|  | 20 | 2018/2/1 | 2018/4/30 | 3 | | 336 | | 1538 | 743.95 | 2.07 | 0.001 |
|  | 21 | 2019/6/1 | 2019/9/30 | 4 | | 1 | | 96 | 7.68 | 12.50 | 0.001 |
|  | 22 | 2021/3/1 | 2021/5/31 | 3 | | 1 | | 88 | 14.16 | 6.22 | 0.001 |
|  | 23 | 2016/3/1 | 2016/4/30 | 2 | | 1 | | 17 | 1.02 | 16.60 | 0.001 |
|  | 24 | 2018/2/1 | 2018/3/31 | 2 | | 1 | | 21 | 2.15 | 9.78 | 0.001 |
|  | **C*** | **Start - End Date** | | | **Duration (Month)** | | **Nº M** | **Observed** | **Expected** | **RR** | **p-value** |
| **Zika** | 1 | 2016/1/1 | 2016/5/31 | | 5 | | 3 | 30791 | 320.07 | 118.37 | 0.001 |
|  | 2 | 2016/1/1 | 2016/5/31 | | 5 | | 1 | 16418 | 9.94 | 1836.15 | 0.001 |
|  | 3 | 2016/1/1 | 2016/4/30 | | 4 | | 297 | 21903 | 296.26 | 85.25 | 0.001 |
|  | 4 | 2016/1/1 | 2016/5/31 | | 5 | | 66 | 13349 | 219.87 | 66.04 | 0.001 |
|  | 5 | 2016/2/1 | 2016/4/30 | | 3 | | 429 | 9582 | 223.42 | 45.51 | 0.001 |
|  | 6 | 2016/1/1 | 2016/5/31 | | 5 | | 106 | 5362 | 302.10 | 18.32 | 0.001 |
|  | 7 | 2016/1/1 | 2016/6/30 | | 6 | | 1 | 3805 | 121.05 | 32.16 | 0.001 |
|  | 8 | 2016/2/1 | 2016/6/30 | | 5 | | 20 | 2760 | 64.66 | 43.40 | 0.001 |
|  | 9 | 2016/1/1 | 2016/5/31 | | 5 | | 141 | 3764 | 228.29 | 16.85 | 0.001 |
|  | 10 | 2016/1/1 | 2016/5/31 | | 5 | | 383 | 3181 | 257.55 | 12.58 | 0.001 |
|  | 11 | 2016/2/1 | 2016/4/30 | | 3 | | 223 | 1789 | 189.81 | 9.52 | 0.001 |
|  | 12 | 2016/1/1 | 2016/4/30 | | 4 | | 2 | 473 | 1.36 | 347.94 | 0.001 |
|  | 13 | 2016/3/1 | 2016/7/31 | | 5 | | 6 | 1465 | 162.38 | 9.09 | 0.001 |
|  | 14 | 2021/4/1 | 2021/10/31 | | 7 | | 1 | 413 | 2.85 | 145.27 | 0.001 |
|  | 15 | 2016/2/1 | 2016/4/30 | | 3 | | 52 | 1114 | 157.92 | 7.10 | 0.001 |
|  | 16 | 2016/3/1 | 2016/5/31 | | 3 | | 10 | 232 | 7.79 | 29.81 | 0.001 |
|  | 17 | 2016/1/1 | 2016/4/30 | | 4 | | 1 | 377 | 43.64 | 8.66 | 0.001 |
|  | 18 | 2016/2/1 | 2016/4/30 | | 3 | | 23 | 237 | 15.54 | 15.27 | 0.001 |
|  | 19 | 2016/3/1 | 2016/5/31 | | 3 | | 79 | 226 | 20.95 | 10.80 | 0.001 |
|  | 20 | 2020/5/1 | 2020/8/31 | | 4 | | 12 | 201 | 18.97 | 10.61 | 0.001 |
|  | 21 | 2016/12/1 | 2017/1/31 | | 2 | | 23 | 96 | 6.70 | 14.34 | 0.001 |
|  | 22 | 2016/2/1 | 2016/3/31 | | 2 | | 149 | 292 | 85.29 | 3.43 | 0.001 |
|  | 23 | 2016/1/1 | 2016/2/29 | | 2 | | 1 | 29 | 0.43 | 68.22 | 0.001 |
|  | 24 | 2016/2/1 | 2016/3/31 | | 2 | | 6 | 32 | 1.19 | 26.92 | 0.001 |
|  | 25 | 2019/3/1 | 2019/7/31 | | 5 | | 1 | 30 | 4.45 | 6.75 | 0.001 |
|  | 26 | 2016/3/1 | 2016/4/30 | | 2 | | 64 | 87 | 39.35 | 2.21 | 0.004 |
|  | 27 | 2016/3/1 | 2016/4/30 | | 2 | | 1 | 8 | 0.27 | 29.17 | 0.021 |

**Table S6.** Space-time multivariate analysis cluster of chikungunya and Zika cases at Brazil, 2015-2021. *C = clusters’ identification number, see Fig. 5c. Nº M = Number of municipalities.

| **C*** | **Start - End Date** | | **Duration (Month)** | **Nº M** | **p-value** | **diseases** | **Observed** | **Expected** | **RR** |
| --- | --- | --- | --- | --- | --- | --- | --- | --- | --- |
| 1 | 2016/3/1 | 2017/7/31 | 17 | 172 | 0.001 | chik | 133318 | 4119.48 | 41.16 |
|  |  |  |  |  |  | Zika | 2500 | 1122.57 | 2.25 |
| 2 | 2016/1/1 | 2016/5/31 | 5 | 1 | 0.001 | chik | 13548 | 33.08 | 418.81 |
|  |  |  |  |  |  | Zika | 16418 | 8.81 | 2073.14 |
| 3 | 2016/1/1 | 2016/6/30 | 6 | 3 | 0.001 | chik | 12740 | 1328.76 | 9.77 |
|  |  |  |  |  |  | Zika | 31660 | 339.43 | 115.52 |
| 4 | 2017/2/1 | 2018/6/30 | 17 | 38 | 0.001 | chik | 21340 | 548.62 | 40.27 |
|  |  |  |  |  |  | Zika | 183 | 144.26 | 1.27 |
| 5 | 2015/11/1 | 2016/7/31 | 9 | 276 | 0.001 | chik | 31129 | 2232.12 | 14.64 |
|  |  |  |  |  |  | Zika | 3770 | 616.02 | 6.24 |
| 6 | 2021/4/1 | 2021/9/30 | 6 | 136 | 0.001 | chik | 23154 | 1399.23 | 17.16 |
|  |  |  |  |  |  | Zika | 1054 | 376.32 | 2.81 |
| 7 | 2016/3/1 | 2016/5/31 | 3 | 249 | 0.001 | chik | 8703 | 710.96 | 12.4 |
|  |  |  |  |  |  | Zika | 2913 | 200.23 | 14.79 |
| 8 | 2021/2/1 | 2021/5/31 | 4 | 13 | 0.001 | chik | 4953 | 43.2 | 115.59 |
|  |  |  |  |  |  | Zika | 25 | 12.32 | 2.03 |
| 9 | 2016/2/1 | 2016/3/31 | 2 | 1 | 0.001 | chik | 3161 | 3.8 | 835.99 |
|  |  |  |  |  |  | Zika | 51 | 1.03 | 49.32 |
| 10 | 2017/2/1 | 2017/8/31 | 7 | 1 | 0.001 | chik | 3487 | 75 | 46.7 |
|  |  |  |  |  |  | Zika | 182 | 21.86 | 8.33 |
| 11 | 2016/1/1 | 2016/6/30 | 6 | 100 | 0.001 | chik | 293 | 1402.06 | 0.21 |
|  |  |  |  |  |  | Zika | 6014 | 401.47 | 15.52 |
| 12 | 2021/3/1 | 2021/4/30 | 2 | 1 | 0.001 | chik | 212 | 0.34 | 625.33 |
|  |  |  |  |  |  | Zika | 3 | 0.082 | 36.71 |
| 13 | 2016/3/1 | 2016/4/30 | 2 | 1 | 0.001 | chik | 17 | 0.91 | 18.74 |
|  |  |  |  |  |  | Zika | 8 | 0.24 | 33.97 |

**Table S7.** Cluster of spatial variation in temporal trends analysis of chikungunya and Zika cases at Brazil, 2015-2021. *Cluster = Clusters’ identification number, see Fig. 6. Nº M = Number of municipalities.

|  | **Cluster** | **Nº M** | **Observed** | **Expected** | **RR** | **Trend (%)** | | **p-value** |
| --- | --- | --- | --- | --- | --- | --- | --- | --- |
|  |  |  |  |  |  | **In** | **Out** |  |
| **chikungunya** | 1 | 15 | 14735 | 12046.92 | 1.23 | 92.06 | -14.76 | 0.001 |
|  | 2 | 246 | 11553 | 14007.47 | 0.82 | 66.10 | -14.34 | 0.001 |
|  | 3 | 245 | 44478 | 23455.54 | 1.97 | 8.54 | -14.98 | 0.001 |
|  | 4 | 25 | 31997 | 10660.68 | 3.11 | 9.16 | -14.53 | 0.001 |
|  | 5 | 165 | 27148 | 14297.00 | 1.94 | 5.11 | -14.19 | 0.001 |
|  | 6 | 63 | 15114 | 15727.19 | 0.96 | 10.33 | -13.92 | 0.001 |
|  | 7 | 89 | 15169 | 17570.69 | 0.86 | 4.30 | -13.80 | 0.001 |
|  | 8 | 198 | 946 | 22358.06 | 0.04 | 44.63 | -13.43 | 0.001 |
|  | 9 | 63 | 1751 | 4187.73 | 0.42 | 23.80 | -13.46 | 0.001 |
|  | 10 | 25 | 1579 | 1632.82 | 0.97 | 17.52 | -13.44 | 0.001 |
|  | 11 | 27 | 7026 | 3371.65 | 2.10 | -0.47 | -13.51 | 0.001 |
|  | 12 | 51 | 243 | 1490.93 | 0.16 | 96.56 | -13.39 | 0.001 |
|  | 13 | 2 | 310 | 68.92 | 4.50 | 65.02 | -13.39 | 0.001 |
|  | 14 | 6 | 238 | 373.99 | 0.64 | 83.94 | -13.39 | 0.001 |
|  | 15 | 5 | 737 | 116.93 | 6.31 | 20.16 | -13.40 | 0.001 |
|  | 16 | 32 | 436 | 2083.10 | 0.21 | 30.65 | -13.39 | 0.001 |
|  | 17 | 2 | 152 | 85.20 | 1.78 | 85.57 | -13.38 | 0.001 |
|  | 18 | 13 | 289 | 502.81 | 0.57 | 36.95 | -13.38 | 0.001 |
|  | 19 | 5 | 728 | 302.39 | 2.41 | 10.08 | -13.39 | 0.001 |
|  | 20 | 113 | 14121 | 7263.69 | 1.97 | -8.62 | -13.48 | 0.001 |
|  | 21 | 1 | 92 | 398.34 | 0.23 | 96.56 | -13.37 | 0.001 |
|  | 22 | 1 | 170 | 78.94 | 2.15 | 37.07 | -13.38 | 0.001 |
|  | 23 | 5 | 92 | 131.55 | 0.70 | 66.99 | -13.37 | 0.001 |
|  | 24 | 1 | 251 | 124.90 | 2.01 | 15.77 | -13.37 | 0.001 |
|  | 25 | 68 | 109 | 2951.92 | 0.04 | 33.95 | -13.37 | 0.001 |
|  | 26 | 107 | 1513 | 6848.76 | 0.22 | -3.48 | -13.39 | 0.001 |
|  | 27 | 39 | 10458 | 10318.14 | 1.01 | -9.91 | -13.42 | 0.001 |
|  | 28 | 1 | 91 | 105.64 | 0.86 | 31.25 | -13.37 | 0.001 |
|  | 29 | 4 | 161 | 167.10 | 0.96 | 14.84 | -13.37 | 0.001 |
|  | 30 | 3 | 848 | 313.91 | 2.70 | -2.11 | -13.38 | 0.001 |
|  | 31 | 2 | 32 | 47.76 | 0.67 | 76.73 | -13.37 | 0.001 |
|  | 32 | 56 | 95 | 1624.73 | 0.06 | 20.73 | -13.37 | 0.001 |
|  | 33 | 5 | 38 | 64.31 | 0.59 | 50.82 | -13.37 | 0.001 |
|  | 34 | 1 | 148 | 74.13 | 2.00 | 9.38 | -13.37 | 0.001 |
|  | 35 | 1 | 288 | 51.29 | 5.62 | 2.28 | -13.37 | 0.001 |
|  | 36 | 169 | 23 | 4618.75 | 0.00 | 70.57 | -13.37 | 0.001 |
|  | 37 | 2 | 113 | 75.52 | 1.50 | 12.64 | -13.37 | 0.001 |
|  | 38 | 1 | 17 | 11.59 | 1.47 | 96.56 | -13.37 | 0.001 |
|  | 39 | 1 | 38 | 93.72 | 0.41 | 36.16 | -13.37 | 0.001 |
|  | 40 | 108 | 208 | 20983.59 | 0.01 | 3.84 | -13.37 | 0.001 |
|  | 41 | 1 | 387 | 99.86 | 3.88 | -1.10 | -13.37 | 0.001 |
|  | 42 | 6 | 397 | 658.38 | 0.60 | -1.30 | -13.37 | 0.001 |
|  | 43 | 1 | 15 | 53.00 | 0.28 | 96.56 | -13.37 | 0.001 |
|  | 44 | 197 | 272 | 23856.17 | 0.01 | 0.85 | -13.37 | 0.001 |
|  | 45 | 22 | 219 | 2120.75 | 0.10 | 2.25 | -13.37 | 0.001 |
|  | 46 | 8 | 61 | 199.15 | 0.31 | 17.61 | -13.37 | 0.004 |
|  | 47 | 7 | 26 | 93.57 | 0.28 | 35.74 | -13.37 | 0.029 |
|  | **Cluster** | **Nº M** | **Observed** | **Expected** | **RR** | **Trend (%)** | | **p-value** |
|  |  |  |  |  |  | **In** | **Out** |  |
| **Zika** | 1 | 397 | 3708 | 6509.07 | 0.56 | 15.92 | -41.64 | 0.001 |
|  | 2 | 168 | 1632 | 4705.27 | 0.34 | 2.77 | -40.60 | 0.001 |
|  | 3 | 33 | 388 | 642.22 | 0.60 | 47.95 | -40.29 | 0.001 |
|  | 4 | 170 | 1413 | 2896.56 | 0.48 | -6.35 | -40.43 | 0.001 |
|  | 5 | 43 | 251 | 1286.82 | 0.19 | 31.51 | -40.20 | 0.001 |
|  | 6 | 178 | 1785 | 5268.60 | 0.33 | -23.35 | -40.29 | 0.001 |
|  | 7 | 71 | 759 | 4017.06 | 0.19 | -15.78 | -40.21 | 0.001 |
|  | 8 | 16 | 92 | 327.90 | 0.28 | 39.42 | -40.12 | 0.001 |
|  | 9 | 231 | 83 | 2620.89 | 0.03 | 44.35 | -40.12 | 0.001 |
|  | 10 | 90 | 2002 | 2626.89 | 0.76 | -27.46 | -40.25 | 0.001 |
|  | 11 | 31 | 100 | 943.34 | 0.11 | 20.15 | -40.12 | 0.001 |
|  | 12 | 37 | 161 | 689.11 | 0.23 | 3.32 | -40.13 | 0.001 |
|  | 13 | 148 | 135 | 2387.61 | 0.06 | 5.61 | -40.12 | 0.001 |
|  | 14 | 3 | 54 | 21.66 | 2.49 | 44.04 | -40.10 | 0.001 |
|  | 15 | 1 | 71 | 40.33 | 1.76 | 13.22 | -40.10 | 0.001 |
|  | 16 | 3 | 174 | 53.44 | 3.26 | -8.34 | -40.12 | 0.001 |
|  | 17 | 9 | 68 | 70.09 | 0.97 | 7.92 | -40.10 | 0.001 |
|  | 18 | 221 | 1946 | 4141.44 | 0.46 | -31.64 | -40.19 | 0.001 |
|  | 19 | 15 | 33 | 159.26 | 0.21 | 32.98 | -40.09 | 0.001 |
|  | 20 | 154 | 1627 | 1836.50 | 0.88 | -32.49 | -40.16 | 0.001 |
|  | 21 | 1 | 18 | 15.60 | 1.15 | 53.03 | -40.09 | 0.001 |
|  | 22 | 1 | 19 | 11.47 | 1.66 | 35.26 | -40.08 | 0.001 |
|  | 23 | 10 | 114 | 271.77 | 0.42 | -15.52 | -40.10 | 0.001 |
|  | 24 | 29 | 126 | 741.99 | 0.17 | -17.58 | -40.10 | 0.001 |
|  | 25 | 1 | 11 | 70.00 | 0.16 | 40.37 | -40.08 | 0.001 |
|  | 26 | 6 | 197 | 83.00 | 2.38 | -25.25 | -40.10 | 0.001 |
|  | 27 | 67 | 140 | 2159.69 | 0.06 | -24.27 | -40.09 | 0.005 |
|  | 28 | 4 | 27 | 40.72 | 0.66 | -5.84 | -40.08 | 0.021 |
